# Preliminary Validation of a High Docosahexaenoic Acid (DHA) and -Linolenic Acid (ALA) Dietary Oil Blend: Tissue Fatty Acid Composition and Liver Proteome Response in Atlantic Salmon (*Salmo salar*) Smolts

**DOI:** 10.1371/journal.pone.0161513

**Published:** 2016-08-24

**Authors:** Waldo G. Nuez-Ortín, Chris G. Carter, Richard Wilson, Ira Cooke, Peter D. Nichols

**Affiliations:** 1 Institute for Marine and Antarctic Studies, University of Tasmania, Private Bag 49, Hobart, TAS 7001, Australia; 2 CSIRO Food Nutrition and Bio-based Products, Oceans & Atmosphere, GPO Box 1538, Hobart, TAS 7001, Australia; 3 Central Science Laboratory, University of Tasmania, Bag 74, Hobart, TAS 7001, Australia; 4 Department of Molecular and Cell Biology, James Cook University, Townsville, QLD 4811, Australia; Universidade de Vigo, SPAIN

## Abstract

Marine oils are important to human nutrition as the major source of docosahexaenoic acid (DHA), a key omega-3 long-chain (≥C_20_) polyunsaturated fatty acid (n-3 LC-PUFA) that is low or lacking in terrestrial plant or animal oils. The inclusion of fish oil as main source of n-3 LC-PUFA in aquafeeds is mostly limited by the increasing price and decreasing availability. Fish oil replacement with cheaper terrestrial plant and animal oils has considerably reduced the content of n-3 LC-PUFA in flesh of farmed Atlantic salmon. Novel DHA-enriched oils with high alpha-linolenic acid (ALA) content will be available from transgenic oilseeds plants in the near future as an alternative for dietary fish oil replacement in aquafeeds. As a preliminary validation, we formulated an oil blend (TOFX) with high DHA and ALA content using tuna oil (TO) high in DHA and the flaxseed oil (FX) high in ALA, and assessed its ability to achieve fish oil-like n-3 LC-PUFA tissue composition in Atlantic salmon smolts. We applied proteomics as an exploratory approach to understand the effects of nutritional changes on the fish liver. Comparisons were made between fish fed a fish oil-based diet (FO) and a commercial-like oil blend diet (fish oil + poultry oil, FOPO) over 89 days. Growth and feed efficiency ratio were lower on the TOFX diet. Fish muscle concentration of n-3 LC-PUFA was significantly higher for TOFX than for FOPO fish, but not higher than for FO fish, while retention efficiency of n-3 LC-PUFA was promoted by TOFX relative to FO. Proteomics analysis revealed an oxidative stress response indicative of the main adaptive physiological mechanism in TOFX fish. While specific dietary fatty acid concentrations and balances and antioxidant supplementation may need further attention, the use of an oil with a high content of DHA and ALA can enhance tissue deposition of n-3 LC-PUFA in relation to a commercially used oil blend.

## 1. Introduction

The importance of seafood in human nutrition and the ability of aquaculture, as it meets the increasing demand for seafood, to provide nutrients traditionally supplied by seafood, particularly key fatty acids, are central issues in global food security [1]. The absolute content of omega-3 longchain (≥C_20_) polyunsaturated fatty aci**d** (n-3 LC-PUFA), particularly docosahexaenoic acid (22:6n-3, DHA) and eicosapentaenoic acid (20:5n-3, EPA), is one of the critical contributions that Atlantic salmon makes to human diet and nutrition [2], and the benefits of an adequate dietary supply of n-3 LC-PUFA are well documented [3, 4]. Current dietary recommendations for weekly intake of n-3 LC-PUFA range between 2 and 14 g [5, 6], equivalent to at least two weekly servings of 100 g of fatty fish such as Atlantic salmon. However, concerns regarding the actual content of n-3 LC-PUFA in the fillet of farmed Atlantic salmon and the potential health benefits delivered to the consumer have been raised [7]. As shown by recent reports from Australia and Europe, current absolute concentrations of the n-3 LC-PUFA have dropped to half (~ 1000 mg per 100 g of muscle) in the last decade and/or show high variability among retailers [2, 8]. This decrease in n-3 LC-PUFA content was attributed by the same authors to industry feeding practices, which are a consequence of reduced fish oil availability, competition from other industries and increasing price. Terrestrial vegetable oils and rendered animal fats are used to replace fish oil in salmon aquafeeds; and although less expensive and more readily available than fish oil, they are low or lacking in n-3 LC-PUFA [2, 9]. Therefore, enhancing the actual nutritional value of Atlantic salmon fillet necessarily requires the inclusion in aquafeeds of new and sustainable oils with higher n-3 LC-PUFA content than those currently in use.

A parallel line of thought is that sustainable feed formulation should also aim to promote efficient tissue deposition of n-3 LC-PUFA. The foundation for this is that tissue fatty acid composition is not only dependent on dietary fatty acid composition, but also on the respective metabolic fates such as utilization for energy, bioconversion or de novo production [10]. The concept of a “n-3 LC PUFA sparing effect” was revised by Codabaccus et al. [11], concluding that equally important to the absolute dietary supply of n-3 LC-PUFA is an increase in the DHA:EPA ratio that promotes enhanced tissue deposition efficiency. Concurrently, the efficiency of tissue deposition of n-3 LC-PUFA in salmonid teleosts can be promoted by increasing the availability of the alpha-linolenic acid (18:3n-3, ALA) precursor, which can be further desaturated and elongated [10, 12, 13], or alternatively, preferentially oxidized over n-3 LC-PUFA if present in high amounts [14, 15].

Over the last 15 years, considerable progress has been made in the field of plant metabolic engineering for effective production of oilseeds rich in n-3 LC-PUFA [16]. This research and development has been encouraged by the relatively low production cost of transgenic oilseeds as well as by the potential capability to adequately scale up for large volume applications [17]. The recent development of a DHA-containing oil extracted from *Camelina sativa* containing fish oil-like DHA levels of 12%, EPA levels of 3% and ALA levels of 29% presents a future alternative to meet the demand for n-3 LC-PUFA from aquaculture [18, 19]. This particular profile suggests promise as a substitute for fish oil because it theoretically supports the idea of enhancing efficiency of tissue deposition of n-3 LC-PUFA by sparing their deposition and promoting of ALA bioconversion. Novel DHA-containing oils extracted from transgenic plants are presently under development [18, 20], are only produced in limited quantities, and at the time of this study were unavailable in sufficient amount to perform a feeding trial. Thus, two oils were blended, tuna oil as a source of DHA (27% of total fatty acids), and flaxseed oil as a source of ALA (56% of total fatty acids), to obtain an oil blend that attempted to mimic the fatty acid profile of a novel *Camelina*-DHA oil. We tested the hypothesis that such a blended oil profile would result in fillet contents of n-3 LC-PUFA higher than those from current alternative oil-based diets and improve the efficiency of tissue deposition of n-3 LC-PUFA as compared to a fish oil-based diet. While EPA-containing oil extracted from transgenic *Camelina* has been produced [21] and tested in Atlantic salmon [22] in the United Kingdom, the present study is the first attempt to perform a preliminary validation of an oil with high DHA and ALA profile in salmon, thereby aiming to assist in the further development and use in aquafeeds of novel DHA-containing oils with high ALA content.

A more precise control of feed composition requires both a broader screening and a more sensitive approach to understanding dietary induced physiological changes in fish. This can be achieved by being able to interpret changes in the liver proteome, as previously reported in response to other dietary modifications [23, 24]. The liver is a crucial site for fatty acid metabolism and body lipid homeostasis in Atlantic salmon and possesses other regulatory functions related to protein and carbohydrate metabolism, immunity or xenobiotic metabolism [22, 25]. With regard to the specific use of sustainable ingredients in salmon aquafeeds, the liver proteome of Atlantic salmon has been studied in response to protein substitution [26], but the relationship between the liver proteome and oil manipulation has not been investigated.

The objective of the present study was to test the effectiveness of an oil blend with high DHA and ALA content, as found in oil from transgenic *Camelina* seeds [18], as a substitute for dietary fish oil in Atlantic salmon. Comparisons were made to a fish oil-based diet and a commercial-like oil blend containing fish oil and poultry oil as is currently used in aquafeeds for Atlantic salmon farmed in Tasmania (Australia). Our findings will primarily contribute towards further development and use of DHA-containing oil from transgenic plants as a sustainable solution for improving the nutritional value of salmon fillet. Secondly, knowledge of the liver proteome response to dietary oil manipulation is likely to provide useful insights into the regulatory mechanisms governing lipid metabolism and other metabolism-relevant mechanisms that directly affect phenotypic traits in Atlantic salmon.

## 2. Materials and Methods

### 2.1. Experimental feeds

Three isonitrogenous and isolipidic experimental feeds were formulated to contain 240 g kg^−1^ of lipid and 490 g kg^−1^ of protein, varying only the lipid source: A 100% fish oil feed (FO), a blend of 20% fish oil and 80% poultry oil (FOPO) regarded as representative of current feeding practices in Tasmania, and a blend of 60% tuna oil and 40% flaxseed oil formulated to mimic the fatty profile of DHA-containing oil from *Camelina* (TOFX) (Table 1). Each feed included fish meal and yttrium oxide as a digestibility marker. Feeds were manufactured into 3 mm diameter pellets using a California Pellet Mill (CL-2, San Francisco, CA, USA), dried and stored at—20°C until use.

**Table 1:** Ingredient and proximate composition of experimental feeds.

	FO	FOPO	TOFX
*Ingredient composition (g kg*^*-1*^*)*			
Fish meal^1^	350	350	350
Casein^2^	146	146	146
Wheat gluten^1^	50	50	50
Soy protein concentrate^1^	116	116	116
Fish oil^3^	190	38	-
Poultry oi^1^	-	152	-
Tuna oil^4^	-	-	114
Flaxseed oil^5^	-	-	76
Starch^6^	74	74	74
Vitamin mineral mix^7^	3	3	3
CMC^8^	10	10	10
Dicalcium Phosphate^9^	6	6	6
Stay-C^7^	1.5	1.5	1.5
Choline chloride^8^	1	1	1
Alpha-celullose^8^	7.5	7.5	7.5
Yttrium oxide^8^	1	1	1
Sipernat^10^	40	40	40
DL-Methionine^11^	4	4	4
*Proximate composition (g kg*^*-1*^ *DM)*			
Dry matter (g kg^-1^)	906.5	897.2	902.0
Crude protein	489.7	492.5	490.2
Total lipid	237.9	247.9	242.5
Ash	111.2	111.0	109.0
Gross energy (MJ kg^-1^)	24.7	24.7	24.6

FO, oil content of feed is 100% fish oil; FOCF, oil content of feed is 20% fish oil and 80% chicken fat; TOFX, oil content of feed is 60% tuna oil and 40% flaxseed oil.

^1^Skretting Australia, Cambridge, TAS, Australia;

^2^MP Biomedicals Australasia Pty., Seven Hills, NSW, Australia;

^3^Chilean anchovy oil, Skretting Australia, Cambridge, TAS, Australia;

^4^Clover Corporation/NuMega Lipids, Melbourne, VIC, Australia;

^5^Kayban, Melbourne, VIC, Australia;

^6^Starch Australasia, Lane Cove, NSW, Australia;

^7^DSM, Wagga, NSW, Australia;

^8^Sigma-Aldrich, Castle Hill, NSW, Australia;

^9^ACE Chemical Company, Adelaide, SA, Australia;

^10^Degussa, Frankfurt, Germany;

^11^BEC Feed Solutions, Goodna, QLD, Australia.

### 2.2. Feeding trial and sampling

The trial was conducted at the Institute for Marine and Antarctic Studies, University of Tasmania (Taroona, Tasmania, Australia). All procedures implemented during this experiment were approved by the University of Tasmania Animal Ethics Committee (Investigation A0014093). Atlantic salmon smolts were sourced from a commercial hatchery (Petuna, Tasmania, Australia), randomly allocated in 12 x 500 L seawater tanks at an initial stocking density of 26 fish tank^−1^ and acclimated for 28 days. Tanks were arranged in two independent six-tank partial recirculation systems, each equipped with a heat-exchanger, protein skimmer, drum filter, UV filter and biological filter. System seawater was continually supplied and progressively replaced twice daily, with tanks supplied at a rate of 8.5 L min^−1^. Tanks were maintained at 15°C water temperature, and at 12 h light:12 h dark photoperiod. Water quality parameters (pH, DO, nitrate and nitrite) were recorded daily and maintained within limits for Atlantic salmon [27]. During acclimation, fish were fed a commercial feed (520 g kg^−1^ of crude protein, 210 g kg^−1^ of fat, and 21.9 MJ kg^−1^ of GE; Skretting, Tasmania, Australia).

At the start of the trial, fish were anaesthetized (Aqui-S^®^ 50 mg L^-1^) [28] and wet weight and fork length measured. A total of 24 fish, representative of the initial population, were euthanized (Aqui-S^®^ 500 mg L^-1^). Twelve whole carcasses were stored at -20°C for initial chemical and fatty acid composition analyses. Dissected liver and dorsal muscle from the other twelve fish were frozen in liquid nitrogen and stored at -80°C for initial proximate and fatty acid composition analyses. Four replicate tanks, two per recirculation system, were randomly assigned to each experimental diet or treatment. Feeds were provided at 1% fish body weight in two daily rations (0900 and 1700 h), and increased uniformly by 0.1% fish body weight every week. Uneaten feed was collected twice daily 10 min after feeding to accurately determine feed intake per tank. At 21-day intervals, all fish were anaesthetized and bulk weighed to monitor growth. After 89 days of growth, eight fish tank^-1^ were euthanized. Four whole carcasses were stored at -20°C for final chemical and fatty acid composition analysis. Dissected dorsal muscle and liver from the other four fish were subsampled, frozen in liquid nitrogen, and stored at -80°C for chemical and fatty acid composition and proteomic analyses. At the end of the trial (day 102), four fish per tank were anaesthetized and stripped for collection of faeces [29] and further proximate and fatty acid composition analyses. Fish were not fed for 24 h prior to being anaesthetized or euthanized.

### 2.3. Chemical composition

Tissues and whole carcasses sampled from the initial population were pooled, whereas tissues, whole carcasses and faeces from the final population were pooled on a per tank basis. Feeds, whole carcasses, tissues and faeces were freeze-dried to constant weight and milled to a fine powder. Dry matter was obtained by drying at 135°C for 2 h and ash content after incineration at 600°C for 2 h [30]. Crude protein was calculated after determination of total nitrogen by Kjeldahl analysis (Kjeltec^™^ 8100, Foss, Denmark), based on N x 6.25 [30]. Total lipid was obtained following overnight extraction using a modified Bligh and Dyer protocol [31], involving a single phase extraction using dichloromethane/methanol/water (1:2:0.8, v/v/v) followed by phase separation to yield a total lipid extract. Gross energy was measured by bomb calorimeter (6725 Semimicro, Parr, IL, USA). Analyses were performed in quadruplicate for experimental diets and initial sampling and in duplicate for final sampling. All analyses were corrected for DM.

### 2.4. Fatty acid analysis

An aliquot of the total lipid extract was trans-methylated in methanol/dichloromethane/hydrochloric acid (10:1:1, v/v/v) at 80°C for 2 h. After addition of mQ water (1 mL), the mixture was extracted with hexane/dichloromethane (4:1, v/v) three times to obtain fatty acid methyl esters (FAME). FAME were made up to a known volume with internal injection standard (19:0 FAME, Nu-Chek Prep, Inc., MN, USA) and analysed by a 7890B gas chromatograph (Agilent Technologies, California, USA) equipped with a Supelco Equity^™^-1 fused silica capillary column (15 m × 0.1 mm i.d., 0.1 μm film thickness), flame ionization detector, split/splitless injector, and a 7683B auto sampler (Agilent Technologies, CA, USA). Helium was used as the carrier gas and samples were injected in splitless mode at an oven temperature of 120°C. After injection, oven temperature was increased to 270°C at 10°C min^-1^ and to a final temperature of 300°C at 5°C min^-1^. Peaks were quantified with ChemStation software (Agilent Technologies, CA, USA) and initially identified using retention times from authentic and laboratory standards. Gas chromatography results are normally subject to an error of up to ±5% of peak area. Absolute and relative values for each detected fatty acid were calculated from the areas of chromatogram peaks.

GC-mass spectrometric (GC-MS) analyses were performed on a Finnigan Trace GC-MS ultra Quadrupole GC-MS (ThermoQuest Trace DSQ, Thermo Electron Corporation, TX, USA). Data was processed with ThermoQuest Xcalibur software (Thermo Electron Corporation, TX, USA). The GC fitted with an on-column injector and a capillary HP-5 Ultra column (50 m x 0.32 mm i.d., 0.17 μm film thickness, Agilent technologies, USA) of similar polarity to that described above. Individual components were identified using mass spectral data and by comparing retention time and MS data with those obtained for authentic and laboratory standards. A full procedural blank analysis was performed concurrent to the sample batch.

### 2.5. Digestibility

Apparent digestibility (AD) was determined by assessing yttrium concentrations in experimental diets and faeces. Yttrium was analysed using inductively coupled plasma mass spectrometry (ELEMENT 2, Thermo Fisher Scientific Inc., MA, USA) following digestion with nitric acid and hydrogen peroxide as previously described [32]. Apparent digestibility coefficients were calculated as (%) = 100 − (100 x (Y_diet_ / Y_faeces_) x (X_faeces_ / X_diet_)), where Y is the percentage of yttrium oxide and X is the percentage of a particular nutrient [33].

### 2.6. Fatty acid mass balance (FAMB)

A whole-body FAMB method was performed on the n-3 biosynthetic pathway to estimate the metabolic fate of ALA as previously described [10].

### 2.7. Liver preparation for proteomic analysis

#### 2.7.1. Protein extraction

Liver tissues from two fish (~ 50 mg each) from each tank (8 treatment^-1^) were individually homogenized in Eppendorf tubes containing lysis buffer (7M urea, 2M thiourea, 50 mM pH 8 Tris) and protease inhibitor cocktail (Roche) using Tissue-Tearor homogenator (Biospec Products, OK, USA). Each extraction was performed for 18–24 h at 4°C with overnight rotation. After removal of insoluble material by centrifugation, an aliquot was precipitated with 100% ethanol (9:1, v/v) overnight. Protein pellets were washed twice in 70% ethanol and re-suspended in lysis buffer. Protein concentrations were estimated with Bradford Protein Assay (Bio-Rad) using plate reader (Synergy TMHT, BioTek, QL, Australia). Sampling pooling was used to reduce the effect of inter-individual variability relative to the biochemical differences between fish groups exposed to different farming conditions [23, 34]. Liver protein extracts were pooled by tank (n = 4) and the volumes adjusted with lysis buffer to achieve a concentration of 1 μg μL^-1^ for each sample pool.

#### 2.7.2. Nano-liquid chromatography and tandem mass spectrometry (LTQ-Orbitrap XL)

Protein samples were trypsin-digested using standard procedures [35] and analyzed by nanoLC-MS/MS using an LTQ-Orbitrap XL and Ultimate 3000 RSLCnano HPLC system (ThermoFisher Scientific, MA, USA). Tryptic peptides (~1 μg) were loaded onto a 20 mm x 75 μm PepMap 100 trapping column (3 μm C_18_) at 5 μl/min, using 98% water, 2% acetonitrile and 0.05% TFA. Peptides were separated at 0.3 μl/min on a 250 mm x 75 μm PepMap 100 RSLC column (2μm C_18_) held at 40°C, using a stepped gradient from 97% mobile phase A (0.1% formic acid in water) to 50% mobile phase B (0.08% formic acid in 80% acetonitrile and 20% water) comprising 3–10% B over 10 min, 10–40% B over 120 min, 40–50% B over 10 min, holding at 95% B for 10 min then re-equilibration in 3% B for 15 min. The LTQ-Orbitrap XL was controlled using Xcalibur 2.1 software in data-dependent mode and MS/MS spectra were acquired as described [35].

#### 2.7.3. Database searching and criteria for protein identification

RAW files from the LTQ-Orbitrap were imported into MaxQuant software version 1.5.1.2 for peptide matching to MS/MS spectra and label-free protein quantification on the basis of median peptide intensity (LFQ) values [36]. MS/MS spectra were searched against the Salmonidae database (http://uniprot.org/taxonomy/8030; 17,795 entries entries) using the Andromeda search engine. Default settings for protein identification were used, including a maximum of two missed cleavages, mass error tolerances of 20 ppm then 4.5 ppm for initial and main peptide searches, respectively, 0.5 Da tolerance for fragment ions, variable oxidation of methionine and fixed carbamidomethylation of cysteine. The false discovery rates for peptide-spectrum matches and protein identification were both set to 0.01. MaxQuant output files of the complete peptide and protein-level mass spectrometry are provided in S1 File.

### 2.8. Calculations and statistical analysis

Standard formulae were used to assess growth, feed efficiency and biometrical data. Specific growth rate was calculated as SGR (% d^-1^) = 100 x (ln Wf / ln Wi) / d, where Wf and Wi are the final and initial weights (g) and d the number of days of the experiment. Feed efficiency ratio (g g^-1^) was determined as FER = W_g_ / FI, where W_g_ is weight gain (g) over the feeding trial and FI is the total feed intake (g). Fulton’s condition factor was calculated as k = W / FL^3^, where W is fish weight (g) and FL is fork length (cm). Hepato-, whole gut-, and pyloric caeca- somatic indices were determined as HSI, GSI, PCSI = (TW / W) x 100, where TW is tissue weight (g) and W is fish weight (g). The % difference in fatty acid concentrations between diet and tissues was calculated as [(dietary fatty acid—tissue fatty acid) / dietary fatty acid] x 100.

Statistical analyses of growth, digestibility and chemical composition were performed using SPSS v22.0 software (IBM Corp., NY, USA). Data interpretation was based on one-way analysis of variance (ANOVA) at a significance level of 0.05. Data were checked with Levene’s test to ensure normality and homogeneity of variance. Where significant differences were detected by ANOVA, data was subjected to Tukey-Kramer HSD post-hoc test. Results were expressed as mean ± standard error (SEM) (n = 4) and different letters within a row were used to denote significant differences (p < 0.05) among treatments.

The effect of two separate recirculation systems as a random factor was explored in the form of a randomized block design, resulting in only a significant difference in terms of feed intake (p_system_ = 0.042, F_system_ = 5.809) and consequently on final weight (p_system_ = 0.004, F_system_ = 15.629), SGR (p_system_ = 0.011, F_system_ = 10.955) and FER (p_system_ = 0.011, F_system_ = 11.006). Noting there was a balanced design and each feed was fed to duplicate tanks in each system. Accordingly, system was computed as random factor within the ANOVA analysis for all variables under study and will not be discussed further.

For statistical analysis of LTQ-Orbitrap mass spectrometry, the “ProteinGroups” output file generated by MaxQuant analysis of liver extracts was analysed in R [37] using the *limma* package [38]. Proteins identified on the basis of a single matching peptide were excluded and only proteins detected in at least three biological replicates in any one treatment group were considered. The effect of diet was investigated by fitting a linear model with log2 protein group intensity as the response and diet and system as explanatory variables. All contrasts between different pairs of diets were extracted from this model. Prior to model fitting, intensity values were normalized using cyclic loess normalization [39] and the method of empirical array quality weights [40] was used to calculate sample reproducibility and down-weight less reproducible samples. After initial model fitting, empirical Bayes [41] was used to calculate moderated test statistics and Benjamini Hochberg correction was applied to adjust p-values for multiple testing. Missing values for all remaining proteins were excluded from the analysis with degrees of freedom adjusted accordingly. Proteins differentially abundant at an adjusted p-value < 0.1 were selected for functional characterization. The salmonidae genes were first mapped to human orthologues using PANTHER [42] and then submitted to STRING v10 [43] for network enrichment analysis.

## 3. Results

### 3.1 Chemical composition of feeds

The three experimental feeds were isonitrogenous (490.8 **±** 0.86 g kg^-1^ DM), isolipidic (242.8 ± 2.89 g kg^-1^ DM) and isoenergetic (24.7 ± 0.03 MJ kg^−1^) (Table 1). The fish oil-based feed (FO) was 2.5 and 2.8-fold higher in DHA and EPA, respectively, than the commercial-like feed (FOPO), resulting in similar DHA:EPA ratios of approximately of 1 (Table 2). The TOFX feed was similar to FO in DHA, but 3.5-fold lower in EPA, resulting in a DHA:EPA ratio of 3.4. The concentration of n-3 LC-PUFA in the TOFX feed was 1.6-fold lower than in the FO feed and 1.7-fold greater than in the FOPO feed. The TOFX feed was highest in ALA, with a concentration 11.4 and 18.7-fold greater than that of the FO and FOPO feeds, respectively. In both FO and TOFX feeds, polyunsaturated fatty acids (PUFA) was the dominant fatty acid group and palmitic acid (16:0, PA) was the dominant fatty acid; in FOPO feed, monounsaturated fatty acids (MUFA) was the dominant fatty acid group with oleic acid (18:1n-9, OA) as the dominant fatty acid.

**Table 2:** Total fatty acid content (mg g^-1^ lipid) and fatty acid composition of oils and feeds (as % total fatty acids).

	Oils				Feeds		
	FO	PO	TO	FX	FO	FOPO	TOFX
*Total fatty acid content*	867.0	838.4	777.4	975.4	852.5	934.7	865.6
*Fatty acid composition*							
14:0	7.5	1.4	3.0	0.0	6.6	2.8	2.1
16:0	17.2	21.3	20.7	5.1	17.5	20.1	15.3
17:0	0.5	0.2	1.3	0.1	0.5	0.3	0.8
18:0	3.4	5.9	5.9	3.7	3.6	5.2	5.1
Other SFA^1^	1.1	0.4	2.3	0.5	1.1	0.6	1.5
Total SFA	29.8	29.2	33.3	9.5	29.3	29.1	24.8
16:1n-7	9.4	6.1	4.6	0.1	8.9	6.7	3.3
18:1n-7	3.2	2.6	2.3	1.0	3.2	2.9	2.0
18:1n-9 (OA)	8.4	41.4	12.6	20.2	8.8	31.0	15.4
20:1n-9	0.8	0.5	1.2	0.3	0.8	0.6	0.8
22:1n-11	0.4	0.0	0.3	0.0	0.4	0.2	0.2
Other MUFA^2^	1.4	0.8	1.5	0.1	1.5	1.1	1.1
Total MUFA	23.6	51.5	22.4	21.7	23.6	42.4	22.8
18:2n-6 (LA)	1.4	11.6	1.2	11.7	2.7	9.5	7.5
18:3n-6	0.3	0.1	0.1	0.0	0.3	0.2	0.1
20:3n6	0.2	0.1	0.1	0.0	0.2	0.1	0.1
20:4n-6 (ARA)	0.8	0.3	1.8	0.0	0.6	0.5	1.1
Other n-6 PUFA^3^	0.7	0.2	2.0	0.1	0.7	0.4	1.3
Total n-6 PUFA	3.2	12.3	5.3	11.7	4.4	10.7	10.0
18:3n-3 (ALA)	0.9	1.9	0.5	56.4	1.1	1.7	18.8
18:4n-3 (SDA)	3.2	0.3	0.6	0.0	2.9	1.0	0.6
20:4n-3 (ETA)	0.8	0.1	0.4	0.0	0.8	0.3	0.3
20:5n-3 (EPA)	16.0	1.5	5.3	0.1	15.6	5.5	4.4
22:5n-3 (DPA)	1.8	0.2	1.2	0.0	1.9	0.8	0.9
22:6n-3 (DHA)	13.5	1.3	27.0	0.0	13.5	5.4	14.7
Total n-3 LC PUFA^4^	32.1	3.2	33.9	0.1	31.7	11.9	20.2
Other n-3 PUFA^5^	0.7	0.1	0.3	0.0	0.7	0.2	0.2
Total n-3 PUFA	36.8	5.5	35.2	56.5	36.4	14.9	39.8
Total PUFA	40.1	17.8	40.6	68.2	40.8	25.6	49.8
n-3:n-6^6^	11.3	0.4	6.6	5.8	8.4	1.4	4.0
DHA:EPA^7^	0.8	0.9	5.1	0.1	0.9	1.0	3.4
ALA:LA^8^	0.6	0.2	0.4	5.8	0.4	0.2	2.5
EPA:ARA^9^	21.1	5.1	2.9	-	27.0	11.0	4.1

Oils: FO, fish oil; PO, poultry oil; TO, tuna oil; FX, flaxseed oil; Feeds: FO, oil content of feed is 100% fish oil; FOPO, oil content of feed is 20% fish oil and 80% poultry oil; TOFX, oil content of feed is 60% tuna oil and 40% flaxseed oil.

Data expressed as mean of four replicates per oil and feed.

^1^Includes 15:0, 20:0, 21:0, 22:0, 23:0 and 24:0.

^2^Includes 16:1n-5, 16:1n-9, 18:1n-5, 20:1n-7, 22:1n-9 and 24:1n-9.

^3^Includes 20:2n-6, 22:5n-6, and 22:4n-6.

^4^Includes 20:4n-3, 20:5n-3, 22:5n-3 and 22:6n-3.

^5^Includes 21:5n-3, 24:6n-3 and 24:5n3.

^6^n-3:n-6 ratio.

^7^DHA:EPA ratio.

^8^ALA:LA ratio.

^9^EPA:ARA ratio.

### 3.2. Growth performance and biometry

Feed intake was similar across treatments and fish fed the three diets doubled their initial wet weight (Table 3). Growth and biometry were not different in FO and FOPO fish, but were negatively affected in TOFX fish. Final weight was significantly lower (p_diet_ = 0.042, F_diet_ = 4.829) in TOFX fish as compared to FO or FOPO fish. SGR (p_diet_ = 0.025, F_diet_ = 6.046) and FER (p_diet_ = 0.009, F_diet_ = 8.893) were significantly lower in TOFX fish. Fork length was not different across treatments, but k was significantly (p_diet_ = 0.033, F_diet_ = 5.420) lower for TOFX fish than for FO or FOPO fish. HSI was not different across treatments. There were significant differences in GSI (p_diet_ = 0.016, F_diet_ = 7.302) and PCSI (p_diet_ = 0.046, F_diet_ = 4.624), with these in TOFX fish not different to those of FOPO fish, but lower than those of FO fish.

**Table 3:** Growth performance, feed utilisation and biometry of Atlantic salmon smolt fed FO, FOPO and TOFX feeds over a 89 day period.

	FO	FOPO	TOFX
Initial weight (g fish^-1^)	104.7 ± 0.83	105.4 ± 1.72	107.0 ± 1.92
Final weight (g fish^-1^)	222.8 ± 5.47 ab	229.3 ± 6.00 a	214.1 ± 5.80 b
Feed intake (g fish^-1^)	130.2 ± 3.22	137.3 ± 5.42	131.4 ± 2.55
SGR^1^ 89d (%)	0.85 ± 0.02 a	0.87 ± 0.04 a	0.78 ± 0.03 b
FER^2^	0.91 ± 0.02 a	0.90 ± 0.03 a	0.81 ± 0.03 b
k^3^	1.1 ± 0.03 a	1.1 ± 0.02 a	1.0 ± 0.01 b
HSI^4^ (%)	1.4 ± 0.17	1.2 ± 0.06	1.3 ± 0.11
GSI^5^ (%)	10.4 ± 0.54 a	9.3 ± 0.21 ab	8.5 ± 0.15 b
PCSI^6^ (%)	4.9 ± 0.25 a	4.4 ± 0.16 ab	4.1 ± 0.11 b

FO, oil content of feed is 100% fish oil; FOPO, oil content of feed is 20% fish oil and 80% poultry oil; TOFX, oil content of feed is 60% tuna oil and 40% flaxseed oil.

Data expressed as mean ± SEM (n = 4). Different letters within a row denotes significant differences among diets as determined by Tukey-Kramer HSD (p<0.05).

^1^Speciific growth rate.

^2^Feed efficiency ratio.

^3^Condition factor.

^4^Hepato-somatic index.

^5^Gut-somatic index.

^6^Pyloric caeca-somatic index.

### 3.3. Digestibility

Dietary oil source did not have a significant effect on apparent digestibility (AD) of dry matter, crude protein, total lipid or gross energy. Digestibility values were on average 69.4 ± 0.09% for dry matter, 89.3 ± 0.32% for crude protein, 94.0 ± 0.08% for lipid, and 80.6 ± 0.93% for gross energy.

AD for fatty acids was significantly affected by dietary oil source, however, differences were smaller than 3% for most individual and classes of fatty acids (S1 Table). AD of DHA was lowest (p_diet_ = 0.004, F_diet_ = 9.993) in TOFX fish, whereas AD of EPA and other n-3 LC-PUFA were not different across treatments. AD of ALA was lowest (p_diet_ = 0.000, F_diet_ = 30.110) in TOFX fish, and the same pattern was also followed for total n-3 PUFA (p_diet_ = 0.002, F_diet_ = 12.780) and total PUFA (p_diet_ = 0.035, F_diet_ = 5.275). AD of total MUFA was higher (p_diet_ = 0.042, F_diet_ = 4.819) in TOFX fish than in FO fish, but not different from FOPO fish, whereas AD of total SFA was not different across treatments and lower than for total PUFA and total MUFA.

### 3.3. Chemical composition of tissues and whole carcasses

Diet did not have a significant effect on chemical composition of tissues and whole carcasses. Contents in white dorsal muscle were on average 256.9 ± 1.74 g kg^-1^ for dry matter, 803.9 ± 7.24 g kg^-1^ DM for crude protein, 98.5 ± 3.49 g kg^-1^ DM for lipid, and 73.0 ± 4.21 g kg^-1^ DM for ash. Contents in liver were on average 258.9 ± 1.17 g kg^-1^ for dry matter, 600.9 ± 7.51 g kg^-1^ DM for crude protein, 153.4 ± 7.10 g kg^-1^ DM for lipid, and 55.6 ± 1.13 g kg^-1^ DM for ash. Contents in whole carcasses were on average 343.1 ± 2.20 g kg^-1^ for dry matter, 498.8.9 ± 7.77 g kg^-1^ DM for crude protein, 414.3 ± 7.52 g kg^-1^ DM for lipid, and 83.9 ± 1.74 g kg^-1^ DM for ash.

### 3.4. Fatty acid composition of tissues

**Fig 1:**
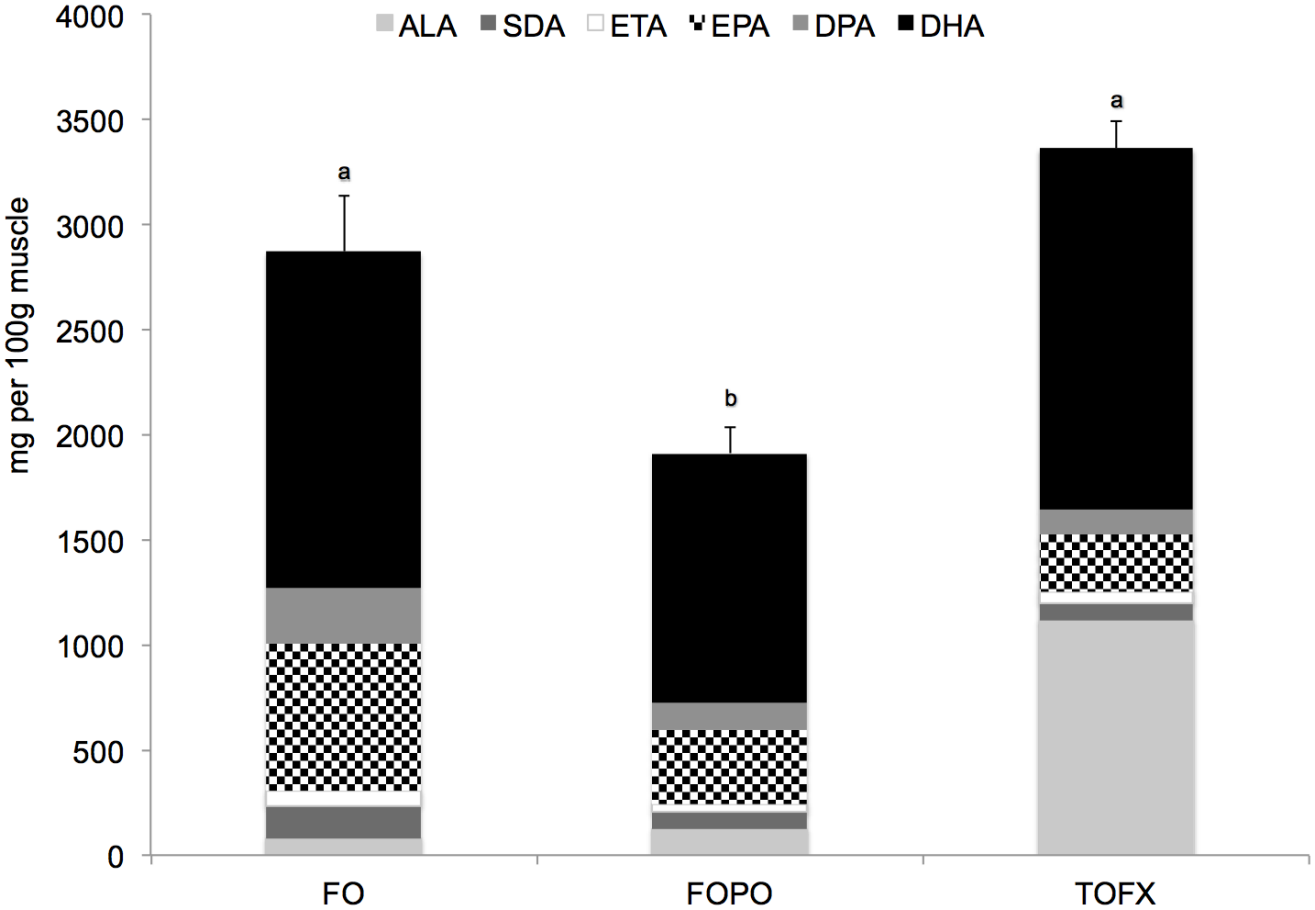
Absolute content of total n-3 PUFA in white dorsal muscle of Atlantic salmon smolt fed FO, FOCF and TOFX feeds over a 89 day period. Data expressed as mg per 100g (dry weight) of tissue. Values are means ± SEM (n = 4). Different letters denote significant differences (p < 0.05) among treatment means.

Fatty acid profiles in muscle tissue were significantly different among dietary treatments (Table 4). The concentration of DHA was not different between FO and TOFX fish, and was higher (p_diet_ = 0.000, F_diet_ = 15.242) than that of FOPO fish. EPA was the highest (p_diet_ = 0.000, F_diet_ = 383.045) in FO fish, and higher in FOPO fish than in TOFX fish. Total n-3 LC-PUFA differed significantly across treatments (p_diet_ = 0.000, F_diet_ = 40.425), with the concentration in TOFX fish being higher than in FOPO fish and lower than in FO fish. ALA was highest (p_diet_ = 0.000, F_diet_ = 2319.401) in TOFX fish and not significantly different between FO and FOPO fish. The concentration of total n-3 PUFA in TOFX fish was higher (p_diet_ = 0.000, F_diet_ = 76.281) than in FOPO fish and was not different from FO fish, and the same pattern was followed in terms of absolute contents (p_diet_ = 0.001, F_diet_ = 15.889) (Fig 1). Total n-6 PUFA did not differ between TOFX and FOPO fish and was higher (p_diet_ = 0.000, F_diet_ = 114.197) than in FO fish. The resulting omega-3:omega-6 (n-3:n-6) ratio was significantly different (p_diet_ = 0.000, F_diet_ = 77.650), being higher in TOFX fish than in FOPO fish and lower than in FO fish. In TOFX and FO fish, PUFA was the dominant fatty acid group with DHA as the dominant fatty acid; in FOPO fish, MUFA was the dominant fatty acid group with OA as the dominant fatty acid.

**Table 4:** Total fatty acid content (mg g^-1^ lipid) and fatty acid composition (% total fatty acid) of dorsal white muscle of Atlantic salmon smolt fed FO, FOCF and TOFX feeds over a 89 day period.

	Initial	FO	FOPO	TOFX
*Total fatty acid content*	871.0 ± 48.81	858.8 ± 31.49	895.8 ± 17.31	882.7 ± 23.68
*Fatty acid composition*				
14:0	2.7 ± 0.07	4.0 ± 0.32 a	2.0 ± 0.14 b	1.5 ± 0.09 b
16:0	15.8 ± 0.05	17.7 ± 0.28 a	18.4 ± 0.30 a	15.2 ± 0.18 b
17:0	0.3 ± 0.00	0.4 ± 0.01 b	0.3 ± 0.00 c	0.6 ± 0.01 a
18:0	5.0 ± 0.06	4.7 ± 0.09 b	5.1 ± 0.05 a	5.3 ± 0.05 a
Other SFA^1^	0.6 ± 0.01	0.7 ± 0.07 b	0.5 ± 0.04 b	0.9 ± 0.03 a
Total SFA	24.3 ± 0.08	27.5 ± 0.41 a	26.2 ± 0.40 a	23.4 ± 0.32 b
16:1n-7	6.0 ± 0.05	6.5 ± 0.34 a	5.3 ± 0.16 b	2.9 ± 0.04 c
18:1n-7	3.3 ± 0.02	3.5 ± 0.07 a	3.3 ± 0.02 b	2.3 ± 0.03 c
18:1n-9 (OA)	29.3 ± 0.38	13.0 ± 0.68 c	28.7 ± 0.72 a	16.9 ± 0.53 b
20:1n-9	1.5 ± 0.02	0.8 ± 0.10 b	1.3 ± 0.03 a	1.0 ± 0.06 b
22:1n-11	0.4 ± 0.01	0.3 ± 0.02 a	0.1 ± 0.01 b	0.2 ± 0.02 b
Other MUFA^2^	1.3 ± 0.02	1.5 ± 0.02 a	1.3 ± 0.01 b	1.2 ± 0.01 c
Total MUFA	42.0 ± 0.42	25.6 ± 0.85 b	40.0 ± 0.90 a	24.4 ± 0.63 b
18:2n-6 (LA)	8.1 ± 0.07	2.9 ± 0.15 c	7.5 ± 0.22 a	6.4 ± 0.06 b
18:3n-6	0.2 ± 0.00	0.1 ± 0.01 b	0.2 ± 0.01 c	0.1 ± 0.01 a
20:3n-6	0.4 ± 0.01	0.3 ± 0.02 b	0.5 ± 0.01 a	0.2 ± 0.01 c
20:4n-6 (ARA)	0.8 ± 0.03	1.0 ± 0.04 ab	0.9 ± 0.07 b	1.1 ± 0.02 a
Other n-6 PUFA^3^	0.9 ± 0.01	0.8 ± 0.04 b	0.9 ± 0.02 b	1.8 ± 0.35 a
Total n-6 PUFA	10.4 ± 0.04	5.0 ± 0.19 b	10.0 ± 0.14 a	9.6 ± 0.35 a
18:3n-3 (ALA)	1.1 ± 0.02	1.0 ± 0.05 b	1.3 ± 0.03 b	12.8 ± 0.23 a
18:4n-3 (SDA)	1.0 ± 0.02	1.9 ± 0.13 a	0.9 ± 0.02 b	1.0 ± 0.05 b
20:4n-3 (ETA)	0.5 ± 0.00	0.8 ± 0.04 c	0.4 ± 0.01 a	0.6 ± 0.03 b
20:5n-3 (EPA)	3.5 ± 0.08	9.0 ± 0.18 a	3.8 ± 0.16 b	3.2 ± 0.11 c
22:5n-3 (DPA)	1.5 ± 0.01	3.3 ± 0.17 a	1.4 ± 0.02 b	1.4 ± 0.02 b
22:6n-3 (DHA)	12.1 ± 0.35	20.8 ± 1.14 a	13.0 ± 1.11 b	19.9 ± 0.85 a
Total n-3 LC PUFA^4^	17.6 ± 0.44	33.9 ± 1.25 a	18.6 ± 1.26 c	25.0 ± 0.90 b
Other n-3 PUFA^5^	0.7 ± 0.01	1.0 ± 0.06 a	0.6 ± 0.07 b	0.6 ± 0.05 b
Total n-3 PUFA	20.5 ± 0.46	37.9 ± 1.16 a	21.3 ± 1.21 b	39.5 ± 0.87 a
Total PUFA	30.9 ± 0.41	43.0 ± 1.05 b	31.3 ± 1.07 c	49.1 ± 0.55 a
n-3:n-6^6^	2.0 ± 0.05	7.6 ± 0.44 a	2.1 ± 0.15 c	4.1 ± 0.20 b
DHA:EPA^7^	3.4 ± 0.02	2.3 ± 0.10 c	3.4 ± 0.18 a	6.2 ± 0.18 b
ALA:LA^8^	0.1 ± 0.00	0.4 ± 0.03 b	0.2 ± 0.01 c	2.0 ± 0.05 a
EPA:ARA^9^	4.4 ± 0.08	9.2 ± 0.52 a	4.5 ± 0.22 b	2.8 ± 0.04 c

FO, oil content of feed is 100% fish oil; FOPO, oil content of feed is 20% fish oil and 80% poultry oil; TOFX, oil content of feed is 60% tuna oil and 40% flaxseed oil.

Data expressed as mean ± SEM (n = 4). Different letters within a row denotes significant differences among diets as determined by Tukey-Kramer HSD (p<0.05).

^1^Includes 15:0, 20:0, 21:0, 22:0, 23:0 and 24:0.

^2^Includes 16:1n-5, 16:1n-9, 18:1n-5, 20:1n-7, 22:1n-9 and 24:1n-9.

^3^Includes 20:2n-6, 22:5n-6, and 22:4n-6.

^4^Includes 20:4n-3, 20:5n-3, 22:5n-3 and 22:6n-3.

^5^Includes 21:5n-3, 24:6n-3 and 24:5n3.

^6^n-3:n-6 ratio.

^7^DHA:EPA ratio.

^8^ALA:LA ratio.

^9^EPA:ARA ratio.

Fatty acid profiles in liver tissue were significantly different among dietary treatments (Table 5). DHA concentration in TOFX fish was not different from those of FO and FOPO fish, but was higher (p_diet_ = 0.029, F_diet_ = 5.564) in FO fish than in FOPO fish. EPA was the highest (p_diet_ = 0.000, F_diet_ = 208.308) in FO fish, and higher in FOPO fish than in TOFX fish. Total n-3 LC-PUFA in TOFX and FOPO fish was not different and was lower (p_diet_ = 0.000, F_diet_ = 37.751) than in FO fish. ALA was highest in TOFX fish and was not significantly different between FO or FOPO fish (p_diet_ = 0.000, F_diet_ = 90.088). Total n-3 PUFA in TOFX fish was higher (p_diet_ = 0.000, F_diet_ = 44.773) than in FOPO fish, but lower than in FO fish. Total n-6 PUFA in TOFX or FOPO fish was not different and lower (p_diet_ = 0.000, F_diet_ = 408.187) than in FO fish. The n-3:n-6 ratio differed significantly across treatments (p_diet_ = 0.000, F_diet_ = 254.544), with TOFX fish being higher than FOPO fish but lower than FO fish. PUFA was the dominant fatty acid group in all dietary treatments with DHA as the dominant fatty acid.

**Table 5:** Total fatty acid content (mg g^-1^ lipid) and fatty acid composition (% total fatty acid) of liver of Atlantic salmon smolt fed FO, FOCF and TOFX feeds over a 89 day period.

	Initial	FO	FOPO	TOFX
*Total fatty acid content*	698.9 ± 15.28	673.7 ± 35.11	676.0 ± 29.07	678.9 ± 8.44
*Fatty acid composition*				
14:0	1.0 ± 0.12	1.9 ± 0.07 b	1.0 ± 0.04 c	0.9 ± 0.07 a
16:0	18.1 ± 0.30	15.9 ± 0.22	14.7 ± 0.21	14.8 ± 0.85
17:0	0.3 ± 0.00	0.4 ± 0.02 b	0.2 ± 0.01 b	0.7 ± 0.07 a
18:0	5.8 ± 0.06	6.8 ± 0.06	6.5 ± 0.18	7.3 ± 0.43
Other SFA^1^	0.4 ± 0.04	0.5 ± 0.02	0.8 ± 0.42	0.7 ± 0.03
Total SFA	25.6 ± 0.48	25.5 ± 0.19	23.3 ± 0.37	24.4 ± 1.31
16:1n-7	3.8 ± 0.07	2.9 ± 0.07 a	2.6 ± 0.05 a	1.7 ± 0.16 a
18:1n-7	3.5 ± 0.05	3.5 ± 0.08 a	3.2 ± 0.04 a	2.4 ± 0.14 a
18:1n-9 (OA)	23.7 ± 0.23	12.1 ± 0.63 a	22.4 ± 0.54 b	15.2 ± 1.33 a
20:1n-9	0.7 ± 0.03	1.4 ± 0.10	2.2 ± 0.18	1.3 ± 0.37
22:1n-11	0.2 ± 0.02	0.1 ± 0.01	0.0 ± 0.01	0.1 ± 0.03
Other MUFA^2^	2.1 ± 0.06	3.2 ± 0.09 b	2.8 ± 0.05 a	2.5 ± 0.09 a
Total MUFA	33.9 ± 0.20	23.2 ± 0.79 b	33.1 ± 0.60 a	23.3 ± 1.79 b
18:2n-6 (LA)	6.5 ± 0.03	1.4 ± 0.03 b	4.2 ± 0.16 a	3.9 ± 0.31 a
18:3n-6	0.3 ± 0.00	0.0 ± 0.00 b	0.1 ± 0.02 a	0.1 ± 0.02 ab
20:3n-6	0.5 ± 0.00	0.4 ± 0.02 b	1.2 ± 0.06 a	0.4 ± 0.04 b
20:4n-6 (ARA)	2.1 ± 0.03	3.4 ± 0.09 ab	3.4 ± 0.02 b	4.0 ± 0.21 a
Other n-6 PUFA^3^	0.8 ± 0.01	0.9 ± 0.06 b	1.4 ± 0.07 ab	2.0 ± 0.22 a
Total n-6 PUFA	10.2 ± 0.04	6.1 ± 0.12 b	10.3 ± 0.15 a	10.3 ± 0.08 a
18:3n-3 (ALA)	0.9 ± 0.07	0.3 ± 0.02 b	0.4 ± 0.03 b	6.0 ± 0.56 a
18:4n-3 (SDA)	0.9 ± 0.02	0.5 ± 0.05	0.3 ± 0.03	0.5 ± 0.09
20:4n-3 (ETA)	0.4 ± 0.00	0.6 ± 0.05 a	0.3 ± 0.02 b	0.7 ± 0.05 a
20:5n-3 (EPA)	4.7 ± 0.04	10.3 ± 0.20 a	5.7 ± 0.28 b	4.8 ± 0.17 c
22:5n-3 (DPA)	1.6 ± 0.02	3.2 ± 0.10 a	1.5 ± 0.10 b	1.4 ± 0.09 b
22:6n-3 (DHA)	19.6 ± 0.31	27.4 ± 0.57 a	23.1 ± 0.59 b	25.1 ± 1.23 ab
Total n-3 LC PUFA^4^	26.2 ± 0.35	41.5 ± 0.68 a	30.7 ± 0.85 b	32.0 ± 1.22 b
Other n-3 PUFA^5^	0.3 ± 0.04	0.4 ± 0.02 a	0.3 ± 0.02 b	0.3 ± 0.02 b
Total n-3 PUFA	28.3 ± 0.36	42.7 ± 0.65 a	31.6 ± 0.83 c	38.8 ± 0.99 b
Total PUFA	38.5 ± 0.32	48.9 ± 0.68 a	41.9 ± 0.84 b	49.1 ± 0.97 a
n-3:n-6^6^	2.8 ± 0.05	7.0 ± 0.16 a	3.1 ± 0.09 c	3.7 ± 0.11 b
DHA:EPA^7^	4.2 ± 0.04	2.7 ± 0.06 c	4.0 ± 0.15 b	5.3 ± 0.31 a
ALA:LA^8^	0.1 ± 0.01	0.2 ± 0.01 b	0.1 ± 0.00 c	1.5 ± 0.05 a
EPA:ARA^9^	2.2 ± 0.04	3.0 ± 0.03 a	1.7 ± 0.08 b	1.2 ± 0.08 c

FO, oil content of feed is 100% fish oil; FOPO, oil content of feed is 20% fish oil and 80% poultry oil; TOFX, oil content of feed is 60% tuna oil and 40% flaxseed oil.

Data expressed as mean ± SEM (n = 4). Different letters within a row denotes significant differences among diets as determined by Tukey-Kramer HSD (p<0.05).

^1^Includes 15:0, 20:0, 21:0, 22:0, 23:0 and 24:0.

^2^Includes 16:1n-5, 16:1n-9, 18:1n-5, 20:1n-7, 22:1n-9 and 24:1n-9.

^3^Includes 20:2n-6, 22:5n-6, and 22:4n-6.

^4^Includes 20:4n-3, 20:5n-3, 22:5n-3 and 22:6n-3.

^5^Includes 21:5n-3, 24:6n-3 and 24:5n3.

^6^n-3:n-6 ratio.

^7^DHA:EPA ratio.

^8^ALA:LA ratio.

^9^EPA:ARA ratio.

**Fig 2:**
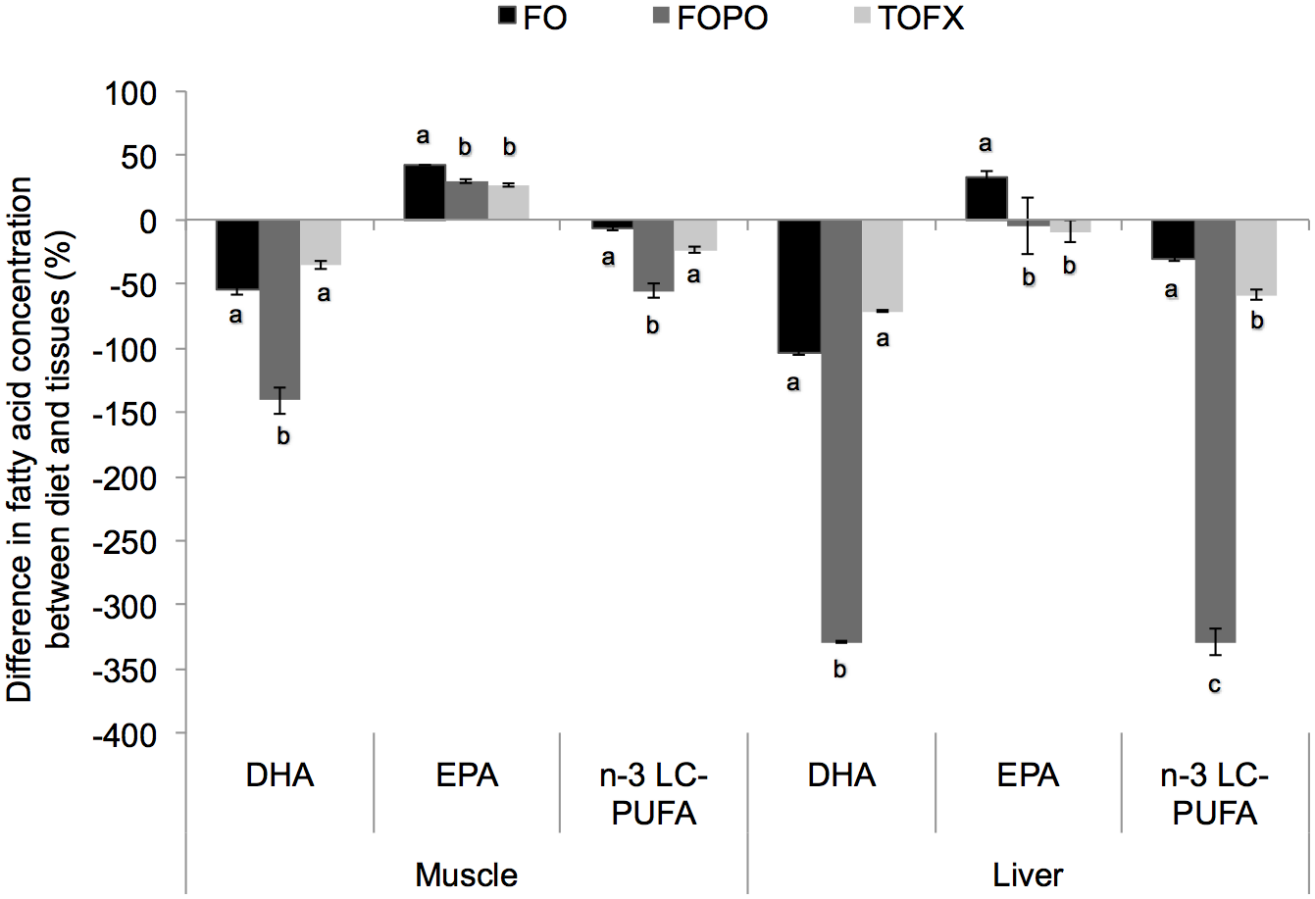
Difference in dietary and tissue (white dorsal muscle and liver) concentrations of EPA, DHA, total n-3 LC-PUFA and ALA. Data expressed as % difference in relation to dietary concentration. Values are means ± SEM (n = 4). Different letters denote significant differences (p < 0.05) among treatment means.

The difference between dietary and tissue concentrations of DHA, EPA, total n-3 LC-PUFA and ALA (in relation to dietary concentration) was significantly different among dietary treatments (Fig 2). The difference between diet and muscle DHA in the TOFX was not different from the FO treatment and was higher (p_diet_ = 0.020, F_diet_ = 16.067) than in the FOPO treatment. The difference between diet and muscle EPA in the TOFX treatment was not different from the FOPO treatment and was lower (p_diet_ = 0.005, F_diet_ = 11.258) than in the FO treatment. For both DHA and EPA, the same pattern of difference observed in muscle was found in liver, with the magnitude of difference being generally larger in liver than in muscle. The difference between diet and muscle n-3 LC-PUFA in the TOFX treatment was higher (p_diet_ = 0.005, F_diet_ = 11.117) than in the FOPO treatment and, although not significant, was numerically lower than in the FO treatment. The difference between diet and liver n-3 LC-PUFA in the TOFX treatment was higher (p_diet_ = 0.000, F_diet_ = 156.978) than in the FOPO treatment and lower than in the FO treatment. The difference between diet and muscle ALA in the TOFX treatment was not different from the FOPO treatment and was higher (p_diet_ = 0.000, F_diet_ = 26.726) than in the FO treatment, whereas the difference between diet and liver ALA did not differ across treatments.

### 3.5. Fatty acid mass balance (FAMB)

FAMB suggested the metabolic fate of ALA was significantly affected by dietary oil source (Table 6). The net intake and absolute disappearance was highest (p_diet_ = 0.000, F_diet_ = 337.219) in TOFX fish. The retention efficiency in TOFX fish was not different from FOPO fish and was lower (p_diet_ = 0.015, F_diet_ = 6.000) than in FO fish. ALA bioconversion was not different across treatments.

**Table 6:** Net intake (μmol fish^-1^) and metabolic fate (μmol fish^-1^ and % total net intake) of ALA in whole carcasses of Atlantic salmon smolt fed FO, FOPO and TOFX feeds over a 89 day period.

	FO	FOPO	TOFX
Net intake^1^	891.4 ± 20.95 c	1677.0 ± 65.43 b	15742.2 ± 363.78 a
Accumulated	818.4 ± 52.28 b	1271.0 ± 170.19 b	9228.0 ± 695.77 a
%	92.0 ± 5.96 a	75.3 ± 8.09 ab	58.4 ± 3.11 b
Disappeared	73.1 ± 54.14 b	406.0 ± 254.52 b	6514.2 ± 356.80 a
%	8.1 ± 6.00 b	24.7 ± 16.17 ab	41.6 ± 3.11 a
Oxidized	0.0 ± 0.00 b	401.5 ± 129.88 b	6401.6 ± 378.75 a
%	0.0 ± 0.00 b	24.5 ± 8.23 a	40.9 ± 3.23 a
Bioconverted	73.1 ± 54.14	4.5 ± 4.46	112.6 ± 24.05
%	8.1 ± 6.00	0.2 ± 0.24	0.7 ± 0.14

FO, oil content of feed is 100% fish oil; FOPO, oil content of feed is 20% fish oil and 80% poultry oil; TOFX, oil content of feed is 60% tuna oil and 40% flaxseed oil.

Data expressed as mean ± SEM (n = 4). Different letters within a row denotes significant differences among diets as determined by Tukey-Kramer HSD (p<0.05).

^1^Calculated as intake minus excretion.

### 3.6. Liver proteomics

**Fig 3:**
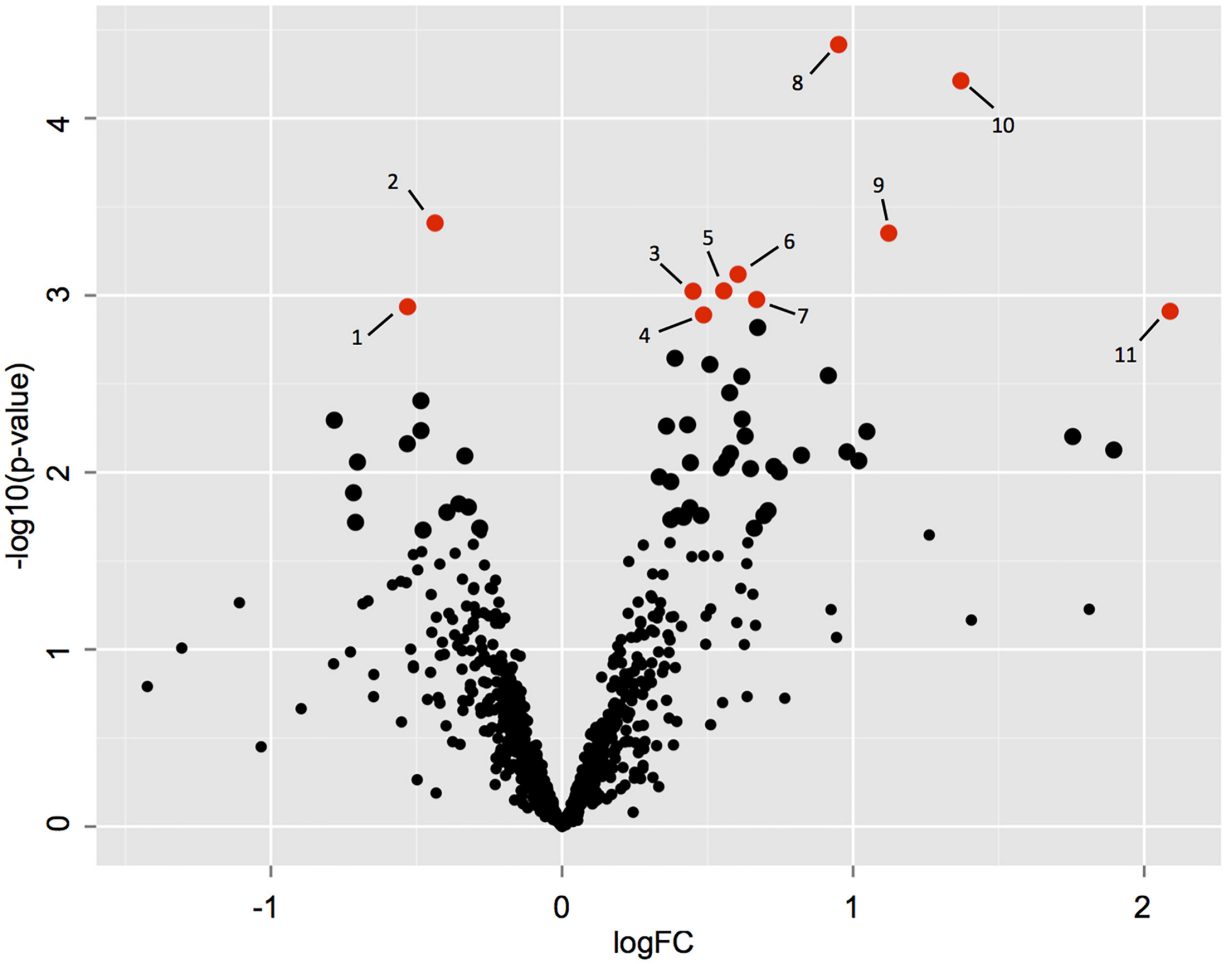
Differences in protein abundance between FO and TOFX livers. Volcano plot displaying differences of the pairwise comparison. Proteins found to be significantly (adjusted p < 0.1; p < 0.05) different between treatments are plotted in red and described in Table 7. Larger black circles represent those proteins significantly different at a lower stringent threshold (adjusted p < 0.3; p < 0.05).

A total of 752 proteins were identified on the basis of two or more unique matching peptide sequences (S2 File). Principal component analysis (PCA) showed maximum separation between TOFX and FO livers whereas FOPO livers showed considerable overlap with both FO and TOFX (S1 Fig). Statistical comparison of intensity values identified significant differences between TOFX and FO livers, the results of which are shown on the volcano plot in Fig 3. On the basis of an adjusted p < 0.1, 11 proteins were differentially abundant, nine of which were up-regulated in TOFX livers and two in FO livers (Table 7). In addition, two proteins were detected only in TOFX livers and one was detected only in FO livers. Fold changes in differentially abundant proteins between FO and TOFX livers ranged from 4.3 to 1.3. Interaction network analysis of differentially abundant proteins revealed a significant enrichment for interacting proteins (p = 0.027) with three interactions (ALDH2-GSTT1, RAB1A-S61A1, S61A1-STT3A).

**Table 7:** Proteins significantly and differentially abundant in liver of Atlantic salmon smolt fed FO and TOFX feeds.

#	Entry^1^	Gene name	Protein name	LFQ intensity^2^ FO	LFQ intensity TOFX	Fold change^3^	No of unique peptides	p-value^4^	Adjusted p-value^5^
1	B5XCS5	ATP5J	ATP synthase-coupling factor 6, mitochondrial	1.21E+06	9.19E+05	-1.3	2	0.001	0.094
2	B5X2T3	ALDH2	Aldehyde dehydrogenase, mitochondrial	7.53E+07	5.40E+07	-1.4	25	0.000	0.089
3	C0HBJ4	RAB1A	Ras-related protein Rab-1A	1.28E+06	1.80E+06	1.4	3	0.001	0.094
4	B5X462	GSTT1	Glutathione S-transferase theta-1	4.55E+06	6.63E+06	1.5	9	0.001	0.094
5	Q68S98		C type lectin receptor A	7.19E+06	1.09E+07	1.5	2	0.001	0.094
6	B5X313	PAHX	Phytanoyl-CoA dioxygenase, peroxisomal	5.37E+06	8.39E+06	1.6	11	0.001	0.094
7	B5DG71	H33	Histone H3	4.69E+07	7.14E+07	1.5	2	0.001	0.094
8	B5X180	UD2A2	UDP-glucuronosyltransferase 2A2	1.23E+06	2.43E+06	2	5	0.000	0.025
9	B5X1I8	STT3A	Dolichyl-diphosphooligosaccharide-protein glycosyltransferase subunit STT3A	7.64E+05	1.76E+06	2.3	5	0.000	0.089
10	B5X322	S61A1	Transport protein Sec61 subunit alpha isoform A	1.01E+06	2.78E+06	2.8	4	0.000	0.025
11	B5X2R4	CP2M1	Cytochrome P450 2M1	1.35E+06	5.59E+06	4.3	12	0.001	0.094
12	B5X970	PNPO	Pyridoxine-5-phosphate oxidase	2.25E+06	ND^6^	-	4	-	-
12	B5DGE7	rtn4	Reticulon	ND	8.42E+05	-	3	-	-
14	C0H9Z9	F13A	Coagulation factor XIII A chain	ND	1.15E+06	-	3	-	-

^1^UniProtKB from UniProt imported Salmo salar database.

^2^Average LFQ intensity of non-normalised values (n = 4).

^3^Fold changes (TOFX vs. FO) in non-normalised LFQ intensities.

^4^p value after pair-wise comparison.

^5^False discovery rate adjustment using Benjamini Hochberg correction. Reported proteins with values less than 0.1.

^6^Not detected.

## 4. Discussion

### 4.1. Fatty acid profiles and nutritional implications

Novel oils extracted from transgenic oilseeds with fish oil-like proportions of DHA may provide the aquafeed industry with an option to enhance the nutritional quality of salmon flesh, as measured by the concentration of n-3 LC-PUFA, to the previous concentrations when marine oils were included in aquafeeds at higher amounts [2]. In the current study, a major comparison was between feeds formulated with fish oil (FO), as a historical standard, a current commercial-like oil blend (FOPO), and a blended oil reflecting the fatty acid profile of DHA-containing *Camelina* oil (TOFX) [18, 19]. The concentration of n-3 LC PUFA in white muscle was used as the most direct indication of how differences in dietary oil composition translated to differences in the product. This reached absolute contents of 2160 mg per 100 g (dry weight) in TOFX fish, and despite not being as high as in FO fish (2760 mg per 100 g), provided an extra 450 mg per 100 g muscle relative to the current industry FOPO diet.

Although the importance of ALA from a health perspective has been generally less reported relative to n-3 LC-PUFA, ALA is receiving interest as a nutraceutical supplement, based on reports of beneficial effects similar to those of n-3 LC-PUFA [44]. ALA is a minor fatty acid in marine oils, however, it is highly and naturally present in *Camelina* and other transgenic oilseeds such as canola, reaching proportions of over 25% of the total fatty acids in oils extracted from transgenic seed [18]. Given the high relative level of ALA in the TOFX oil (22.8% total fatty acids), the muscle content of ALA was the highest in TOFX fish, with absolute levels of 1110 mg per 100 g of muscle in comparison with 86 and 123 mg per 100g of muscle in FO and FOPO fish, respectively. ALA is an essential fatty acid (EFA) for humans, and intake recommendations have been established at a minimum of 14 g weekly [45].

Some evidence suggests that a balanced dietary n-3:n-6 ratio is positive in maintaining optimal human health [46, 47]. A ratio of 1:1–2 has been proposed as a target for adult human nutrition in relation to ratios of 1:10–20 in current western diets [48]. Consumption of fatty fish, and salmon in particular, is advised in western societies for the health benefits associated with high n-3 LC-PUFA content [5, 6]. The intake of these, and n-3 in general rather than n-6, largely control the human dietary n-3:n-6 ratio [49]. The n-3:n-6 ratio in TOFX fish (4.4), albeit not being as high as in FO fish (7.6), was markedly higher than in FOPO fish (2.2). This was directly attributed to the greater accumulation of both n-3 LC-PUFA and ALA, and implies that aquafeeds with high DHA and ALA content would enhance the nutritional quality of farmed salmon, and consequently the potential health benefits to the consumer relative to commercial blends containing a high proportion of poultry oil.

In aiming to enhance the current and future nutritional value of salmon, fish nutritionists should maximize deposition of n-3 LC-PUFA [15]. In the present study, the pattern of tissue deposition in relation to diet was explored by the difference in the relative composition between diet and tissue as previously reported by Codabaccus et al. [11]. In both muscle and liver, the difference in terms of DHA was similar between TOFX and FO treatments, whereas the difference in terms of EPA was lower in TOFX treatment than in FO treatment. This comparison indicates enhanced overall efficiency of tissue deposition of n-3 LC-PUFA in the TOFX treatment, as shown by the lower difference between dietary and tissue n-3 LC-PUFA in relation to FO; though the dissimilarity in this difference was only numerical in muscle, it was significant in liver. These results were consistent with our hypothesis and with Codabaccus et al. [11], with the two studies collectively demonstrating that increasing the DHA:EPA ratio in relation to a fish-oil based diet promotes a tissue deposition “sparing effect”of n-3 LC-PUFA. This “sparing effect” is based on the mechanism of selective conservation of DHA; whereas n-3 LC-PUFA are normally oxidized for energy production if supplied in surplus, DHA is preferentially deposited as EPA is more extensively oxidized [11, 50, 51]. Accordingly, the DHA:EPA ratio of tissues increased in relation to diet, with this increase being less pronounced in TOFX fish than in FO fish due to the lower amount of EPA available for oxidation. Equally important, but unrelated to the DHA:EPA ratio, the sparing effect on n-3 LC-PUFA is also determined by absolute dietary concentration values as well as by the abundance of other SFA and MUFA that are more readily catabolized [11, 14, 50]. This was clearly observed in FOPO treatment, which, despite a dietary DHA:EPA ratio similar to that of FO diet (~ 1), showed the lowest difference between dietary and tissue n-3 LC-PUFA concentration and consequently the highest efficiency in tissue deposition. Low n-3 LC-PUFA in FOPO diet promoted minimum EPA oxidation and maximum DHA deposition, with the more abundant oleic acid (18:1n-9, OA) and linoleic acid (18:2n-6, LA) in FOPO being used for energy production and thereby likely contributing to enhanced sparing and resultant tissue deposition of n-3 LC-PUFA.

Atlantic salmon hepatocytes possess the ability to desaturate and elongate C_18_ fatty acids such as ALA to the long-chain and more unsaturated fatty acids [52–55]. Hence, we evaluated whether a high ALA concentration, as that occurring in the TOFX diet, might contribute to enhancing tissue deposition of n-3 LC-PUFA in relation to the FO diet by promoting desaturation and elongation. ALA in TOFX fish was largely accumulated and/or oxidized, and only minimally (< 1%) bioconverted, and thus no further ALA bioconversion was observed in relation to FO or FOPO fish. Limited or negligible bioconversion of ALA to n-3 LC-PUFA has been attributed to high availability of either substrate (ALA) or end-product (DHA). Whereas excessive C_18_ fatty acids can affect elongation activities by limiting the ability of delta-6-desaturase to act on C_24_ and consequently restraining DHA production [56, 57], the high abundance of n-3 LC-PUFA, particularly DHA rather than EPA, can limit their possible biosynthesis due to an inhibitory effect on delta-6-desaturase and Elovl-2-like elongase enzymes [58–60]. ALA bioconversion has been reported to reach 25% of total net intake in Atlantic salmon fed a diet with full replacement of fish oil with *Camelina* oil [13, 55]; although ALA concentration was similar to that of TOFX diet (~ 20% total fatty acid), DHA was approximately 10-fold lower than in TOFX diet. Taken together, these findings suggest that DHA is likely more limiting than ALA itself in promoting ALA bioconversion in Atlantic salmon fed high DHA and ALA diets. The fairly high concentration of LA in the TOFX diet (7.5% total fatty acids) could also evoke competition between LA and ALA as substrates for the delta-6-desaturase enzyme, as proposed by composition-based studies in teleost [61]. However, this possibility is unlikely for salmonids, where more specific FAMB studies have shown that bioconversion of ALA is favored over LA due to the greater affinity of delta-6-desaturase for the n-3 pathway [12, 62]. In addition to bioconversion, an alternative pathway for ALA to improve tissue deposition of n-3 LC PUFA is through beta-oxidation. ALA has a high rate of oxidation and, if present in large proportions, it is more readily utilized for energy production than other fatty acids [14, 50, 63]. For this hypothesis to be tested, a different approach using graded levels of ALA and constant levels of n-3 LC-PUFA and DHA:EPA ratio would be more suitable.

### 4.2. Growth and performance

Important commercial factors in the feeding of diets containing new alternative oils to Atlantic salmon also relate to the effects they have on growth performance. Several types of alternative oils have been investigated in salmon feeds, including vegetable [51, 55, 63–65], animal fats [9, 66] as well as new DHA and EPA containing oils [22, 67]. These studies have collectively shown that growth is typically unaffected by dietary lipid source provided EFA requirements are met. All diets in the present study were formulated with non-defatted fishmeal that contained sufficient n-3 LC-PUFA to satisfy the EFA requirements of salmon [68]. No difference in growth performance was found between FO fish and FOPO fish, as observed in previous studies with Atlantic salmon where fish oil was replaced at 50–75% with poultry oil [9, 11]. However, TOFX diet had a small but negative effect on growth performance, feed efficiency, condition factor as well as in gut- and pyloric caeca- somatic indexes. Feed intake and digestibility of the main nutrients were not affected by dietary treatment, thus the observed detrimental effects were rather attributed to metabolic alterations resulting from impaired nutrient utilization and associated to the specific fatty acid composition of the TOFX diet. In this respect, n-3 LC PUFA increase beta-oxidation in white fat preventing adipocyte hypertrophy and lipid accumulation [69, 70], and this effect is further supported by the adipogenic capacity of ALA [71, 72]. This offers a possible explanation for the observed lower gut somatic indices observed in TOFX fish and that could be the result of repressed visceral fat deposition.

### 4.3. Liver proteomics—potential oxidative stress responses

The liver proteome provides an informative biological matrix to study diet-induced changes and better understand the physiological basis of different phenotypic traits [23, 24]. Overall, our quantitative comparison among the liver proteomes found the most significantly different proteins between TOFX and FO diets, whereas comparisons between FOPO and FO, and between FOPO and TOFX, revealed no significant differences. This is consistent with the fact that TOFX and FO livers showed maximum separation by PCA analysis, whereas FOPO livers showed considerable overlap with both FO and TOFX.

Several proteins related to protein biogenesis in the ER and secretory protein trafficking were among the more highly up-regulated proteins in the TOFX livers, such as the alpha subunit of Sec6 (S61A1) and the glycosyltransferase subunit STT3A (STT3A). These two proteins were also increased in TOFX livers relative to FOPO livers, albeit at a less stringent cut-off (adjusted p < 0.3). However, the majority of protein alterations in the liver of TOFX fish were related to oxidative stress and detoxification pathways, such as changes in glutathione S-transferase theta-1 (GSTT1), cytochrome P450 2M1 (CP2M1) and mitochondrial aldehyde dehydrogenase (ALDH2). GSTT1 catalyzes the conjugation of the reduced form of glutathione to xenobiotic substrates and up-regulation has been associated to detoxification and increased protection against reactive molecules causing oxidative stress, such as hydrogen peroxides or aldehydes generated from lipid peroxidation [73, 74]. Glutathione transferases operate in parallel with aldehyde dehydrogenases and cytochrome P450 as hepato-protective mechanism against oxidative stress [75], and thus alterations of these proteins have been observed in fish in response to temperature-induced [76] and xenobiotic-induced [77, 78] oxidative stress. ALDH2 was down-regulated in TOFX livers, following the trend previously reported in response to lipid peroxidation [79, 80] and/or to the presence of hydrogen peroxide [81, 82]. In turn, expression of CP2M1 was increased, corroborating the role of cytochrome P450 as compensatory mechanism when the aldehyde dehydrogenase pathway is compromised [75].

Lipid peroxidation-induced oxidative stress was also supported by changes in UD2A2 and pyridoxine-5-phosphate oxidase (PNPO). UD2A2 is the precursor of UDP-glucuronosyl transferase and up-regulation in TOFX fish may be related to the role of UDP-glucuronosyl transferase and glutathione transferases in preventing the propagation of lipid peroxidation [83]. PNPO catalyzes several reactions in the metabolism of vitamin B6, which has an antioxidant role by inhibiting lipid peroxidation or by serving as a coenzyme in the glutathione antioxidant defense system [84]. Increased oxidative stress has been reported in vitamin B6 deficient animals [85], suggesting that down-regulation (to below detection limits) of PNPO in TOFX livers may have contributed to this response. Lipid peroxidation in TOFX livers was also evident from changes in the abundance of several additional proteins (indicated by the data points labeled on Fig 3), albeit at a less stringent threshold for statistical significance (adjusted p < 0.3). Proteins that participate in the process of fatty acid oxidation (ECHA, NCPR, ECPH and PAHX) and in the subsequent antioxidant response (PRDX5, CATA and HBA), as well as oxidoreductases (CRYL1, AL3A2, G6PD) that catalyze these oxidative reactions, were mostly detected at elevated levels in the TOFX diet. Also related to fatty acid oxidation was the up-regulation of PAYH. This protein, along with AL3A2, catalyze the alpha-oxidation of phytanic acid, which is commonly found in high concentrations in tuna oil [86, 87], thus reflecting the specific use of tuna oil in TOFX diet.

The alterations observed in the liver proteome of TOFX fish were indicative of oxidative stress related to peroxidative damage. This may be attributed to the fatty acid formulation of TOFX diet and related therefore to the impaired performance observed in fish. A possible cause was the higher total PUFA in TOFX diet, as evoked from the lower digestibility in relation to the other two diets, and from the lower tissue concentration in relation to feed. Increased susceptibility of fish to oxidative damage has been associated with excessive dietary PUFA; this relation is dependent on the efficacy of the antioxidant system [88–90], and on the proportion that PUFA represents in the total dietary lipid [91, 92]. In the present experiment, all feeds were considered on the safe side in terms of protection from peroxidation; feeds were evenly supplemented with antioxidant (vitamin C supplied by Stay-C, and vitamin E contained in the vitamin premix) in comparable amounts to those used in previous studies testing feeds with similar n-3 LC-PUFA content [22, 88], and stored at -20°C. The PUFA concentration in TOFX diet were the highest across treatment and of 42% of the total lipid, lying within the range (~ 37%–50% total lipid) of previous data in salmonids that showed increased susceptibility to peroxidation through to growth reduction [91, 93, 94]. In a more recent study with Atlantic salmon fed EPA-containing oil (ECO) extracted from transgenic *Camelina*, higher PUFA concentration (~55% of total lipid) did not affect growth [22], suggesting that fish vulnerability to oxidative damage is dependent on both the quality of the input oil(s), total PUFA and the balance among specific PUFA. While the formulation of TOFX and ECO diets were similar in terms of oil inclusion and nutrient composition, the most noticeable difference between the two were the high content of DHA and ALA (TOFX diet) versus a high content of EPA and LA (ECO diet). High dietary DHA amounts may induce oxidative stress and cause adverse effects on fish growth [90]. The threshold from which such responses can be triggered in fish is broad (~ 10%–36% total lipid) and correlated to the respective balance with the antioxidant supply [88–90, 95]. DHA concentration in TOFX diet was high (12.7% total lipid), but close to those of the FO diet (11.5% total lipid), thus high DHA as unique cause of oxidative stress in TOFX fish was rather unlikely. In contrast, ALA concentration in TOFX diet was considerably high (~ 16% of total lipid) in relation to the other diets and not far from dietary concentrations (~ 19%– 25% total lipid) that have previously resulted in growth detriment in Atlantic salmon [55, 96]. The negative effects of high dietary ALA have been associated with an increased susceptibility to lipid peroxidation and immunosuppression by a reduction in the antioxidant capacity provided by alpha-tocopherol [97–99]. We therefore speculate that the antioxidant defense in TOFX fish could have been overwhelmed by the high dietary PUFA concentration, and in particular by the combination of high DHA and ALA. Despite the dietary inclusion of antioxidants, it is possible that the high susceptibility of DHA to oxidative damage was enhanced in our study by ALA-induced depression of the antioxidant system. The increased liver detoxification activities and small reduction in performance probably relates to these mechanisms. In this line, DHA is also more susceptible than EPA to oxidative breakdown [100]. The observed impaired performance in fish fed the TOFX diet in relation to those fed the EPA-containing oil diet, as in Betancor et al. [22], could be explained by the higher susceptibility of fish on the TOFX diet to oxidative damage and the need of extra antioxidant supplementation when fed oils rich in DHA and ALA. Future feeding trials should therefore consider the use of higher inclusion of antioxidants in feeds using such an oil blend, although considerably higher levels of antioxidants are present in *Camelina* and canola oils.

## 4.4. Conclusion

We used an oil blend with similar fatty acid composition to that of a DHA-containing oil extracted from transgenic *Camelina* [18] and applied liver proteomics as an approach to gain insight into the future potential use of DHA-containing oils extracted from transgenic seeds, which are presently under development and limited in quantity. Such an oil blend (TOFX) clearly enhanced both the muscle content of n-3 LC-PUFA, DHA in particular, and the n-3:n-6 ratio as compared to a current commercial blend oil (FOPO) diet. From the human consumer perspective, DHA-containing oils extracted from transgenic terrestrial oil seeds are therefore potentially suitable as a means to improve the nutritional quality for consumers of salmon fillet. The particular fatty acid profile of the TOFX oil also promoted a more efficient deposition of n-3 LC-PUFA in muscle and liver in relation to a fish oil-based diet; this was attributed to less inefficient utilization, in particular as occurs via beta-oxidation, of n-3 LC-PUFA due to the higher DHA:EPA ratio, rather than to bioconversion from the high**er** content of the ALA precursor. The TOFX oil triggered an oxidative stress response that was likely associated to impaired growth. This draws attention to the necessity of considering the balance between total and specific PUFA and antioxidant levels present in the diet and the possible need for supplementation in order to protect PUFA from oxidative damage in both the feed and in the fish. Notwithstanding, we also note that unlike the oil blend used in this study, the pending transgenic terrestrial oils do contain elevated levels of naturally occurring antioxidants including carotenes and tocopherols (CSIRO, unpublished data). This aspect will need further consideration for the future inclusion of DHA-containing oils extracted from transgenic *Camelina* or canola in salmon aquafeeds.

The mass spectrometry proteomics data have been deposited to the ProteomeXchange Consortium via the PRIDE partner repository with the dataset identifier PXD004114

## Supporting Information

S1 Fig Principal component analysis (PCA) of the liver proteome profiles. Data points are marked with sample identifiers and replicate number.
(TIF)

S1 File MaxQuant output files of the complete peptide and protein-level mass spectrometry. (XLSX)

S2 File List of proteins identified and proteins showing significant (adjusted p-value 0.1–0.3) fold changes. (XLSX)

S1 Table Apparent digestibility (AD; %) of fatty acids in Atlantic salmon smolt fed FO, FOPO and TOFX diets over a 89 day period. (DOCX)

## Abbreviations

n-3 LC-PUFA: omega-3 long-chain (≥C_20_) polyunsaturated fatty acids
DHA: docosahexaenoic acid
EPA: eicosapentaenoic acid
ALA: alpha-linolenic acid
LA: linoleic acid
OA: oleic acid
PUFA: polyunsaturated fatty acids
n-3: omega-3
n-6: omega-6
n-3:n:6 ratio: omega-3:omega-6 ratio
AD: apparent digestibility
FAMB: fatty acid mass balance

## References

[1] Watson RA, Nowara GB, Hartmann K, Green BS, Tracey SR, Carter CG. Marine foods sourced from farther as their use of global ocean primary production increases. Nat Commun. 2015;6 10.1038/ncomms8365 PMC4490567 26079714

[2] Nichols P, Glencross B, Petrie J, Singh S. Readily available sources of long-chain omega-3 oils: Is farmed Australian seafood a better source of the good oil than wild-caught seafood? Nutrients. 2014;6(3):1063–79. 10.3390/nu6031063 24618601 PMC3967178

[3] Delgado-Lista J, Perez-Martinez P, Lopez-Miranda J, Perez-Jimenez F. Long chain omega-3 fatty acids and cardiovascular disease: a systematic review. Br J Nutr. 2012;107(SupplementS2):S201–S13. 10.1017/S0007114512001596 22591894

[4] Bell GA, Kantor ED, Lampe JW, Kristal AR, Heckbert SR, White E. Intake of long-chain omega-3 fatty acids from diet and supplements in relation to mortality. Am J Epidemiol. 2014;179(6):710–20. Epub 2014/02/06. 10.1093/aje/kwt326 24496442 PMC3939849

[5] EFSA. Scientific Opinion on Dietary Reference Values for fats, including saturated fatty acids, polyunsaturated fatty acids, monounsaturated fatty acids, trans fatty acids, and cholesterol. EFSA Journal. 2010;8:1461.

[6] NHMRC. Nutrient Reference Values for Australia and New Zealand. Canberra, Australia: NHMRC, 2006.

[7] Fry JP, Love DC, MacDonald GK, West PC, Engstrom PM, Nachman KE, et al Environmental health impacts of feeding crops to farmed fish. Environ Int. 2016;91:201–14. 10.1016/j.envint.2016.02.022. 10.1016/j.envint.2016.02.022 26970884

[8] Henriques J, Dick JR, Tocher DR, Bell JG. Nutritional quality of salmon products available from major retailers in the UK: content and composition of n-3 long-chain PUFA. Br J Nutr. 2014;112(6):964–75. Epub 2014/07/16. 10.1017/s0007114514001603 . 25017007

[9] Emery JA, Smullen RP, Turchini GM. Tallow in Atlantic salmon feed. Aquaculture. 2014;422–423(0):98–108. 10.1016/j.aquaculture.2013.12.004.

[10] Turchini GM, Francis DS. Fatty acid metabolism (desaturation, elongation and beta-oxidation) in rainbow trout fed fish oil- or linseed oil-based diets. Br J Nutr. 2009;102(1):69–81. Epub 2009/01/07. 10.1017/s0007114508137874 . 19123959

[11] Codabaccus BM, Carter CG, Bridle AR, Nichols PD. The “n−3 LC-PUFA sparing effect” of modified dietary n−3 LC-PUFA content and DHA to EPA ratio in Atlantic salmon smolt. Aquaculture. 2012;356–357(0):135–40. 10.1016/j.aquaculture.2012.05.024.

[12] Emery JA, Hermon K, Hamid NKA, Donald JA, Turchini GM. Δ-6 Desaturase substrate competition: Dietary linoleic acid (18:2n-6) has only trivial effects on α-linolenic acid (18:3n-3) bioconversion in the teleost rainbow trout. PLoS One. 2013;8(2). . 23460861 10.1371/journal.pone.0057463 PMC3583879

[13] Hixson SM, Parrish CC, Anderson DM. Use of camelina oil to replace fish oil in diets for farmed salmonids and Atlantic cod. Aquaculture. 2014;431(43–52). 10.1016/j.aquaculture.2014.04.042.

[14] Budge SM, Penney SN, Lall SP. Response of tissue lipids to diet variation in Atlantic salmon (Salmo salar): Implications for estimating diets with fatty acid analysis. J Exp Mar Biol Ecol. 2011;409(1–2):267–74. 10.1016/j.jembe.2011.09.002.

[15] Sanden M, Stubhaug I, Berntssen MH, Lie O, Torstensen BE. Atlantic salmon (Salmo salar L.) as a net producer of long-chain marine omega-3 fatty acids. J Agric Food Chem. 2011;59(23):12697–706. Epub 2011/10/25. 10.1021/jf203289s . 22017199

[16] Ruiz-Lopez N, Usher S, Sayanova OV, Napier JA, Haslam RP. Modifying the lipid content and composition of plant seeds: engineering the production of LC-PUFA. Appl Microbiol Biotechnol. 2015;99(1):143–54. Epub 2014/11/25. 10.1007/s00253-014-6217-2 25417743 PMC4286622

[17] Petrie JR, Singh SP. Expanding the docosahexaenoic acid food web for sustainable production: engineering lower plant pathways into higher plants. AoB Plants. 2011;2011:plr011 Epub 2011/01/01. 10.1093/aobpla/plr011 22476481 PMC3114564

[18] Petrie JR, Shrestha P, Belide S, Kennedy Y, Lester G, Liu Q, et al Metabolic Engineering *Camelina sativa* with Fish Oil-Like Levels of DHA. PLoS One. 2014;9(1):1–9. 10.1371/journal.pone.0085061 PMC3897407 24465476

[19] Petrie JR, Shrestha P, Zhou XR, Mansour MP, Liu Q, Belide S, et al Metabolic engineering plant seeds with fish oil-like levels of DHA. PLoS One. 2012;7(11):e49165 Epub 2012/11/13. 10.1371/journal.pone.0049165 23145108 PMC3492320

[20] Kitessa SM, Abeywardena M, Wijesundera C, Nichols PD. DHA-containing oilseed: a timely solution for the sustainability issues surrounding fish oil sources of the health-benefitting long-chain omega-3 oils. Nutrients. 2014;6(5):2035–58. Epub 2014/05/27. 10.3390/nu6052035 24858407 PMC4042577

[21] Ruiz-Lopez N, Haslam RP, Napier JA, Sayanova O. Successful high-level accumulation of fish oil omega-3 long-chain polyunsaturated fatty acids in a transgenic oilseed crop. The Plant Journal. 2014;77(2):198–208. 10.1111/tpj.12378 24308505 PMC4253037

[22] Betancor MB, Sprague M, Usher S, Sayanova O, Campbell PJ, Napier JA, et al A nutritionally-enhanced oil from transgenic *Camelina sativa* effectively replaces fish oil as a source of eicosapentaenoic acid for fish. Sci Rep. 2015;5 10.1038/srep08104 http://www.nature.com/srep/2015/150129/srep08104/abs/srep08104.html-supplementary-information. PMC4309969 25632018

[23] Ghisaura S, Anedda R, Pagnozzi D, Biosa G, Spada S, Bonaglini E, et al Impact of three commercial feed formulations on farmed gilthead sea bream (Sparus aurata, L.) metabolism as inferred from liver and blood serum proteomics. Proteome Sci. 2014;12:44 10.1186/s12953-014-0044-3. PMC4200174. 25342931 PMC4200174

[24] Rodrigues PM, Silva TS, Dias J, Jessen F. Proteomics in aquaculture: applications and trends. J Proteomics. 2012;75:4325–45. 10.1016/j.jprot.2012.03.042 22498885

[25] Monroig O, Zheng X, Morais S, Leaver MJ, Taggart JB, Tocher DR. Multiple genes for functional 6 fatty acyl desaturases (Fad) in Atlantic salmon (Salmo salar L.): gene and cDNA characterization, functional expression, tissue distribution and nutritional regulation. Biochim Biophys Acta. 2010;1801(9):1072–81. Epub 2010/04/21. 10.1016/j.bbalip.2010.04.007 . 20403458

[26] Sissener NH, Martin SA, Cash P, Hevroy EM, Sanden M, Hemre GI. Proteomic profiling of liver from Atlantic salmon (Salmo salar) fed genetically modified soy compared to the near-isogenic non-GM line. Mar Biotechnol (NY). 2010;12(3):273–81. Epub 2009/07/21. 10.1007/s10126-009-9214-1 . 19618241

[27] Wedemeyer GA. Physiology of fish in intensive culture systems. New York: Chapman and Hall; 1996.

[28] Javahery S, Nekoubin H, Moradlu AH. Effect of anaesthesia with clove oil in fish (review). Fish Physiol Biochem. 2012;38:1545–52. 10.1007/s10695-012-9682-5 22752268

[29] Percival SB, Lee PS, Carter CG. Validation of a technique for determining apparent digestibility in large (up to 5 kg) Atlantic salmon (Salmo salar L.) in seacages. Aquaculture. 2001;201:315–27.

[30] AOAC. Official methods of analysis. 16th ed Washington, DC: AOAC International; 1995.

[31] Alhazzaa R, Bridle AR, Nichols PD, Carter CG. Replacing dietary fish oil with Echium oil enriched barramundi with C18 PUFA rather than long-chain PUFA. Aquaculture. 2011;312(1–4):162–71. 10.1016/j.aquaculture.2010.12.023.

[32] Ward DA, Carter CG, Townsend AT. The use of yttrium oxide and the effect of faecal collection timing for determining the apparent digestibility of minerals and trace elements in Atlantic salmon (Salmo salar, L.) feeds. Aquacult Nutr. 2005;11(1):49–59. 10.1111/j.1365-2095.2004.00323.x

[33] Maynard LALJK. Animal nutrition. New York: McGraw-Hill; 1969.

[34] Melis R, Cappuccinelli R, Roggio T, Anedda R. Addressing marketplace gilthead sea bream (Sparus aurata L.) differentiation by 1H NMR-based lipid fingerprinting. Food Res Int. 2014;63, Part B(0):258–64. 10.1016/j.foodres.2014.05.041.

[35] Wilson R, Golub SB, Rowley L, Angelucci C, Karpievitch YV, Bateman JF, et al Novel elements of the chondrocyte stress response identified using an in vitro model of mouse cartilage degradation. J Proteome Res. 2016;4(15):1033–50. 10.1021/acs.jproteome.5b01115 26794603

[36] Cox J, Hein MY, Luber CA, Paron I, Nagaraj N, Mann M. Accurate proteome-wide label-free quantification by delayed normalization and maximal peptide ratio extraction, termed MaxLFQ. Mol Cell Proteomics. 2014;13(9):2513–26. Epub 2014/06/20. 10.1074/mcp.M113.031591 24942700 PMC4159666

[37] R Core Team. R: A language and environment for statistical computing. R Foundation for Statistical Computing Vienna, Austria 2015.

[38] Ritchie ME, Phipson B, Wu D, Hu Y, Law CW, Shi W, et al limma powers differential expression analyses for RNA-sequencing and microarray studies. Nucleic Acids Res. 2015 10.1093/nar/gkv007 PMC4402510 25605792

[39] Bolstad BM, Irizarry RA, Astrand M, Speed TP. A comparison of normalization methods for high density oligonucleotide array data based on variance and bias. Bioinformatics. 2003;19(2):185–93. Epub 2003/01/23. . 12538238 10.1093/bioinformatics/19.2.185

[40] Ritchie ME, Diyagama D, Neilson J, van Laar R, Dobrovic A, Holloway A, et al Empirical array quality weights in the analysis of microarray data. BMC Bioinformatics. 2006;7:261 Epub 2006/05/23. 10.1186/1471-2105-7-261 16712727 PMC1564422

[41] Smyth GK. Linear models and empirical bayes methods for assessing differential expression in microarray experiments. Stat Appl Genet Mol Biol. 2004;3: Article3. Epub 2006/05/02. 10.2202/1544-6115.1027 . 16646809

[42] Mi H, Muruganujan A, Thomas PD. PANTHER in 2013: modeling the evolution of gene function, and other gene attributes, in the context of phylogenetic trees. Nucleic Acids Res. 2013;41(Database issue):D377–86. Epub 2012/11/30. 10.1093/nar/gks1118 23193289 PMC3531194

[43] Szklarczyk D, Franceschini A, Wyder S, Forslund K, Heller D, Huerta-Cepas J, et al STRING v10: protein-protein interaction networks, integrated over the tree of life. Nucleic Acids Res. 2015;43(Database issue):D447–52. Epub 2014/10/30. 10.1093/nar/gku1003 25352553 PMC4383874

[44] Blondeau N. The nutraceutical potential of omega-3 alpha-linolenic acid in reducing the consequences of stroke. Biochimie. 2016;120:49–55. Epub 2015/06/21. 10.1016/j.biochi.2015.06.005 . 26092420

[45] Colquhoun D, Ferreira-Jardim A, Udell T, Eden B. Review of evidence: Fish, fish oils and n-3 polyunsaturated fatty acids and cardiovascular health. National Heart Foundation of Australia, 2008.

[46] Simopoulos AP. The importance of the omega-6/omega-3 fatty acid ratio in cardiovascular disease and other chronic diseases. Exp Biol Med (Maywood). 2008;233(6):674–88. Epub 2008/04/15. 10.3181/0711-mr-311 . 18408140

[47] Williams CD, Whitley BM, Hoyo C, Grant DJ, Iraggi JD, Newman KA, et al A high ratio of dietary n-6/n-3 polyunsaturated fatty acids is associated with increased risk of prostate cancer. Nutr Res. 2011;31(1):1–8. Epub 2011/02/12. 10.1016/j.nutres.2011.01.002 . 21310299

[48] Simopoulos AP. Evolutionary aspects of diet: the omega-6/omega-3 ratio and the brain. Mol Neurobiol. 2011;44(2):203–15. Epub 2011/02/01. 10.1007/s12035-010-8162-0 . 21279554

[49] Deckelbaum RJ. n-6 and n-3 Fatty Acids and Atherosclerosis: Ratios or Amounts? Atertio Thromb Vasc Biol. 2010;30(12):2325–6. 10.1161/atvbaha.110.214353 21084701

[50] Stubhaug I, Lie Ø, Torstensen BE. Fatty acid productive value and β-oxidation capacity in Atlantic salmon (*Salmo salar L*.) fed on different lipid sources along the whole growth period. Aquacult Nutr. 2007;13(2):145–55. 10.1111/j.1365-2095.2007.00462.x

[51] Bransden MP, Carter CG, Nichols PD. Replacement of fish oil with sunflower oil in feeds for Atlantic salmon (*Salmo salar L*.): effect on growth performance, tissue fatty acid composition and disease resistance. Comp Biochem Physiol B Biochem Mol Biol. 2003;135(4):611–25. Epub 2003/08/02. . 12892753 10.1016/s1096-4959(03)00143-x

[52] Tocher DR, Bell JG, Dick JR, Crampton VO. Effects of dietary vegetable oil on Atlantic salmon hepatocyte fatty acid desaturation and liver fatty acid compositions. Lipids. 2003;38(7):723–32. Epub 2003/09/26. . 14506835 10.1007/s11745-003-1120-y

[53] Stubhaug I, Tocher DR, Bell JG, Dick JR, Torstensen BE. Fatty acid metabolism in Atlantic salmon (*Salmo salar L*.) hepatocytes and influence of dietary vegetable oil. Biochim Biophys Acta. 2005;1734(3):277–88. Epub 2005/06/01. 10.1016/j.bbalip.2005.04.003 . 15921956

[54] Zheng X, Torstensen BE, Tocher DR, Dick JR, Henderson RJ, Bell JG. Environmental and dietary influences on highly unsaturated fatty acid biosynthesis and expression of fatty acyl desaturase and elongase genes in liver of Atlantic salmon (*Salmo salar*). Biochim Biophys Acta. 2005;1734(1):13–24. 10.1016/j.bbalip.2005.01.006. 15866479

[55] Hixson SM, Parrish CC, Anderson DM. Full substitution of fish oil with camelina (*Camelina sativa*) oil, with partial substitution of fish meal with camelina meal, in diets for farmed Atlantic salmon (*Salmo salar*) and its effect on tissue lipids and sensory quality. Food Chem. 2014;157(0):51–61. 10.1016/j.foodchem.2014.02.026. 24679751

[56] Thanuthong T, Francis DS, Manickam E, Senadheera SD, Cameron-Smith D, Turchini GM. Fish oil replacement in rainbow trout diets and total dietary PUFA content: II) Effects on fatty acid metabolism and in vivo fatty acid bioconversion. Aquaculture. 2011;322–323(0):99–108. 10.1016/j.aquaculture.2011.09.026.

[57] Senadheera SD, Turchini GM, Thanuthong T, Francis DS. Effects of dietary alpha-linolenic acid (18:3n-3)/linoleic acid (18:2n-6) ratio on fatty acid metabolism in Murray cod (*Maccullochella peelii peelii*). Journal of Agriculture and Food Chemistry. 2011;59(3):1020–30. Epub 2011/01/13. 10.1021/jf104242y . 21222433

[58] Thomassen MS, Rein D, Berge GM, Østbye T-K, Ruyter B. High dietary EPA does not inhibit Δ5 and Δ6 desaturases in Atlantic salmon (*Salmo salar L*.) fed rapeseed oil diets. Aquaculture. 2012;360–361(0):78–85. 10.1016/j.aquaculture.2012.07.001.

[59] Zheng XZ, Tocher DR, Dickson CA, Bell JG, Teale AJ. Effects of diets containing vegetable oil on expression of genes involved in highly unsaturated fatty acid biosyn- thesis in liver of Atlantic salmon (*Salmo salar*). Aquaculture. 2004;236(1–4):467–83.

[60] Glencross B, De Santis C, Bicskei B, Taggart J, Bron J, Betancor M, et al A comparative analysis of the response of the hepatic transcriptome to dietary docosahexaenoic acid in Atlantic salmon (*Salmo salar*) post-smolts. BMC Genomics. 2015;16(1):1–12. 10.1186/s12864-015-1810-z 26345987 PMC4562122

[61] Vagner M, Santigosa E. Characterization and modulation of gene expression and enzymatic activity of delta-6 desaturase in teleosts: A review. Aquaculture. 2011;315(1–2):131–43. 10.1016/j.aquaculture.2010.11.031.

[62] Thanuthong T, Francis DS, Senadheera SP, Jones PL, Turchini GM. LC-PUFA biosynthesis in rainbow trout is substrate limited: use of the whole body fatty acid balance method and different 18:3n-3/18:2n-6 ratios. Lipids. 2011;46(12):1111–27. Epub 2011/09/06. 10.1007/s11745-011-3607-4 . 21892784

[63] Bell JG, Henderson RJ, Tocher DR, McGhee F, Dick JR, Porter A, et al Substituting fish oil with crude palm oil in the diet of Atlantic Salmon (*Salmo salar*) affects muscle fatty acid composition and hepatic fatty acid metabolism. J Nutr. 2002;132(2):222–30. 11823582 10.1093/jn/132.2.222

[64] Bell JG, Henderson RJ, Tocher DR, Sargent JR. Replacement of dietary fish oil with increasing levels of linseed oil: modification of flesh fatty acid compositions in Atlantic salmon (*Salmo salar*) using a fish oil finishing diet. Lipids. 2004;39(3):223–32. Epub 2004/07/06. . 15233400 10.1007/s11745-004-1223-5

[65] Bell JG, McEvoy J, Tocher DR, McGhee F, Campbell PJ, Sargent JR. Replacement of fish oil with rapeseed oil in diets of Atlantic salmon (*Salmo salar*) affects tissue lipid compositions and hepatocyte fatty acid metabolism. J Nutr. 2001;131(5):1535–43. Epub 2001/05/08. . 11340112 10.1093/jn/131.5.1535

[66] Emery JA, Smullen R, Keast RSJ, Turchini GM. Viability of tallow inclusion in Atlantic salmon diet, as assessed by an on-farm grow out trial. Aquaculture. 2016;451:289–97. 10.1016/j.aquaculture.2015.09.023.

[67] Miller MR, Nichols PD, Carter CG. Replacement of fish oil with thraustochytrid Schizochytrium sp. L oil in Atlantic salmon parr (*Salmo salar L*) diets. Comp Biochem Physiol A Mol Integr Physiol. 2007;148(2):382–92. Epub 2007/06/26. 10.1016/j.cbpa.2007.05.018 . 17588797

[68] NRC. Nutrient requirements of fish and shrimp: The National Academies Press; 2011.

[69] Parrish CC, Pathy DA, Angel A. Dietary fish oils limit adipose tissue hypertrophy in rats. Metabolism. 1990;39(3):217–9. Epub 1990/03/01. . 2308514 10.1016/0026-0495(90)90038-e

[70] Todorčević M, Vegusdal A, Gjøen T, Sundvold H, Torstensen BE, Kjær MA, et al Changes in fatty acids metabolism during differentiation of Atlantic salmon preadipocytes; Effects of n-3 and n-9 fatty acids. Biochim Biophys Acta. 2008;1781(6–7):326–35. 10.1016/j.bbalip.2008.04.014. 10.1016/j.bbalip.2008.04.014 18503782

[71] Zhou X, Wu W, Chen J, Wang X, Wang Y. AMP-activated protein kinase is required for the anti-adipogenic effects of alpha-linolenic acid. Nutr Metab (Lond). 2015;12:10 Epub 2015/03/17. 10.1186/s12986-015-0006-5 25774202 PMC4358912

[72] Oliva ME, Ferreira MR, Chicco A, Lombardo YB. Dietary Salba (Salvia hispanica L) seed rich in alpha-linolenic acid improves adipose tissue dysfunction and the altered skeletal muscle glucose and lipid metabolism in dyslipidemic insulin-resistant rats. Prostaglandins Leukot Essent Fatty Acids. 2013;89(5):279–89. Epub 2013/10/15. 10.1016/j.plefa.2013.09.010 . 24120122

[73] Pajaud J, Kumar S, Rauch C, Morel F, Aninat C. Regulation of signal transduction by glutathione transferases. Int J Hepatol. 2012;2012:11 10.1155/2012/137676 PMC3474235 23094162

[74] Singh S, Brocker C, Koppaka V, Ying C, Jackson B, Matsumoto A, et al Aldehyde dehydrogenases in cellular responses to oxidative/electrophilic stress. Free Radic Biol Med. 2013;56:89–101. 23195683 10.1016/j.freeradbiomed.2012.11.010 PMC3631350

[75] Amunom I, Dieter LJ, Tamasi V, Cai J, Conklin DJ, Srivastava S, et al Cytochromes P450 catalyze the reduction of alpha,beta-unsaturated aldehydes. Chem Res Toxicol. 2011;24(8):1223–30. Epub 2011/07/20. 10.1021/tx200080b 21766881 PMC3180908

[76] Ibarz A, Martin-Perez M, Blasco J, Bellido D, de Oliveira E, Fernandez-Borras J. Gilthead sea bream liver proteome altered at low temperatures by oxidative stress. Proteomics. 2010;10(5):963–75. Epub 2010/02/05. 10.1002/pmic.200900528 . 20131326

[77] Moore MJ, Mitrofanov IV, Valentini SS, Volkov VV, Kurbskiy AV, Zhimbey EN, et al Cytochrome P4501A expression, chemical contaminants and histopathology in roach, goby and sturgeon and chemical contaminants in sediments from the Caspian Sea, Lake Balkhash and the Ily River Delta, Kazakhstan. Mar Pollut Bull. 2003;46(1):107–19. Epub 2003/01/22. . 12535976 10.1016/s0025-326x(02)00325-9

[78] Lee RF, Anderson JW. Significance of cytochrome P450 system responses and levels of bile fluorescent aromatic compounds in marine wildlife following oil spills. Mar Pollut Bull. 2005;50(7):705–23. 10.1016/j.marpolbul.2005.04.036. 15946701

[79] Hjelle JJ, Petersen DR. Hepatic aldehyde dehydrogenases and lipid peroxidation. Pharmacol Biochem Behav. 1983;18 Suppl 1:155–60. Epub 1983/01/01. . 6634832 10.1016/0091-3057(83)90164-8

[80] Wenzel P, Hink U, Oelze M, Schuppan S, Schaeuble K, Schildknecht S, et al Role of reduced lipoic acid in the redox regulation of mitochondrial aldehyde dehydrogenase (ALDH-2) activity. Implications for mitochondrial oxidative stress and nitrate tolerance. J Biol Chem. 2007;282(1):792–9. Epub 2006/11/15. 10.1074/jbc.M606477200 . 17102135

[81] Towell JF 3rd, Wang RI. Hydrogen peroxide-induced glutathione depletion and aldehyde dehydrogenase inhibition in erythrocytes. Biochem Pharmacol. 1987;36(13):2087–93. Epub 1987/07/01. . 3038114 10.1016/0006-2952(87)90135-3

[82] Oelze M, Knorr M, Schell R, Kamuf J, Pautz A, Art J, et al Regulation of human mitochondrial aldehyde dehydrogenase (ALDH-2) activity by electrophiles in vitro. J Biol Chem. 2011;286(11):8893–900. 10.1074/jbc.M110.190017 21252222 PMC3058968

[83] Olatunde Farombi E. Mechanisms for the hepatoprotective action of Kolaviron: Studies on hepatic enzymes, microsomal lipids and lipid peroxidation in carbontetrachloride-treated rats Pharmacol Res. 2000;42(1):75–80. 10.1006/phrs.1999.0648. 10860638

[84] Kannan K, Jain SK. Effect of vitamin B6 on oxygen radicals, mitochondrial membrane potential, and lipid peroxidation in H2O2-treated U937 monocytes. Free Radic Biol Med. 2004;36(4):423–8. Epub 2004/02/21. 10.1016/j.freeradbiomed.2003.09.012 . 14975445

[85] Hsu C-C, Cheng C-H, Hsu C-L, Lee W-J, Huang S-C, Huang Y-C. Role of vitamin B6 status on antioxidant defenses, glutathione, and related enzyme activities in mice with homocysteine-induced oxidative stress. 2015 2015;59 Epub 2015-01-27. 10.3402/fnr.v59.25702 PMC4417078 25933612

[86] Jansen GA, Wanders RJA. Alpha-Oxidation. Biochim Biophys Acta. 2006;1763(12):1403–12. 10.1016/j.bbamcr.2006.07.012. 16934890

[87] van den Brink DM, van Miert JNI, Dacremont G, Rontani J-F, Jansen GA, Wanders RJA. Identification of fatty aldehyde dehydrogenase in the breakdown of phytol to phytanic acid. Mol Genet Metab. 2004;82(1):33–7. 10.1016/j.ymgme.2004.01.019 15110319

[88] Kjær MA, Todorčević M, Torstensen BE, Vegusdal A, Ruyter B. Dietary n-3 HUFA affects mitochondrial fatty acid β-oxidation capacity and susceptibility to oxidative stress in Atlantic salmon. Lipids. 2008;43(9):813–27. 10.1007/s11745-008-3208-z 18615261

[89] ØStbye TK, Kjær MA, Rørå AMB, Torstensen B, Ruyter B. High n-3 HUFA levels in the diet of Atlantic salmon affect muscle and mitochondrial membrane lipids and their susceptibility to oxidative stress. Aquacult Nutr. 2011;17(2):177–90. 10.1111/j.1365-2095.2009.00721.x

[90] Betancor MB, Almaida-Pagán PF, Sprague M, Hernández A, Tocher DR. Roles of selenoprotein antioxidant protection in zebrafish, *Danio rerio*, subjected to dietary oxidative stress. Fish Physiol Biochem. 2015:1–16. 10.1007/s10695-015-0040-2 25750091

[91] Olsen RE, Løvaas E, Lie Ø. The influence of temperature, dietary polyunsaturated fatty acids, α-tocopherol and spermine on fatty acid composition and indices of oxidative stress in juvenile Arctic char, *Salvelinus alpinus* (*L*.). Fish Physiol Biochem. 1999;20(1):13–29. 10.1023/A:1007767827996

[92] Glencross BD. Exploring the nutritional demand for essential fatty acids by aquaculture species. Rev Aquaculture. 2009;1(2):71–124. 10.1111/j.1753-5131.2009.01006.x

[93] Yu TC, Sinnhuber RO. Effects of dietary ω3 and ω6 fatty acids on growth and feed conversion efficiency of coho salmon (*Oncorhynchus kisutch*). Aquaculture. 1979;16:31–8.

[94] Olsen RE, Henderson RJ. Muscle fatty acid composition and oxidative stress indices of Arctic charr, *Salvelinus alpinus* (*L*.), in relation to dietary polyunsaturated fatty acid levels and temperature. Aquacult Nutr. 1997;3(4):227–38. 10.1046/j.1365-2095.1997.00091.x

[95] Stéphan G, Guillaume J, Lamour F. Lipid peroxidation in turbot (*Scophthalmus maximus*) tissue: effect of dietary vitamin E and dietary n − 6 or n − 3 polyunsaturated fatty acids. Aquaculture. 1995;130(2–3):251–68. 10.1016/0044-8486(94)00322-F.

[96] Ruyter, RØsjØ, Einen, Thomassen. Essential fatty acids in Atlantic salmon: effects of increasing dietary doses of n-6 and n-3 fatty acids on growth, survival and fatty acid composition of liver, blood and carcass. Aquacult Nutr. 2000;6(2):119–27. 10.1046/j.1365-2095.2000.00137.x.

[97] McGuire SO, Alexander DW, Fritsche KL. Fish oil source differentially affects rat immune cell alpha-tocopherol concentration. J Nutr. 1997;127(7):1388–94. Epub 1997/07/01. . 9202096 10.1093/jn/127.7.1388

[98] Wu D, Meydani SN, Meydani M, Hayek MG, Huth P, Nicolosi RJ. Immunologic effects of marine- and plant-derived n-3 polyunsaturated fatty acids in nonhuman primates. Am J Clin Nutr. 1996;63(2):273–80. Epub 1996/02/01. . 8561071 10.1093/ajcn/63.2.273

[99] Schimke I, Haberland A, Wirth M, Papies B, Moritz V, Baumann G. Influence of long-term supplementation with alpha-linolenic acid on myocardial lipid peroxidation and antioxidative capacity in spontaneously hypertensive rats. Prostaglandins Leukot Essent Fatty Acids. 1997;57(6):545–50. Epub 1998/02/07. . 9431820 10.1016/s0952-3278(97)90558-5

[100] Song JH, Fujimoto K, Miyazawa T. Polyunsaturated (n-3) fatty acids susceptible to peroxidation are increased in plasma and tissue lipids of rats fed docosahexaenoic acid-containing oils. J Nutr. 2000;130(12):3028–33. Epub 2000/12/09. . 11110863 10.1093/jn/130.12.3028

