# Correction: Preliminary Validation of a High Docosahexaenoic Acid (DHA) and α-Linolenic Acid (ALA) Dietary Oil Blend: Tissue Fatty Acid Composition and Liver Proteome Response in Atlantic Salmon (*Salmo salar*) Smolts

**DOI:** 10.1371/journal.pone.0197800

**Published:** 2018-05-17

**Authors:** The PLOS ONE Staff

The title appears incorrectly. The publisher apologizes for the error. The correct title is: Preliminary Validation of a High Docosahexaenoic Acid (DHA) and α-Linolenic Acid (ALA) Dietary Oil Blend: Tissue Fatty Acid Composition and Liver Proteome Response in Atlantic Salmon (*Salmo salar*) Smolts. The correct citation is Nuez-Ortín WG, Carter CG, Wilson R, Cooke I, Nichols PD (2016) Preliminary Validation of a High Docosahexaenoic Acid (DHA) and α-Linolenic Acid (ALA) Dietary Oil Blend: Tissue Fatty Acid Composition and Liver Proteome Response in Atlantic Salmon (*Salmo salar*) Smolts. PLoS ONE 11(8): e0161513. https://doi.org/10.1371/journal.pone.0161513
