# Supplementary figures and images for: Preliminary Validation of a High Docosahexaenoic Acid (DHA) and -Linolenic Acid (ALA) Dietary Oil Blend: Tissue Fatty Acid Composition and Liver Proteome Response in Atlantic Salmon (Salmo salar) Smolts
Source: PLoS One. 2016 Aug 24;11(8):e0161513. doi: 10.1371/journal.pone.0161513 (PMC4996530; doi:10.1371/journal.pone.0161513)

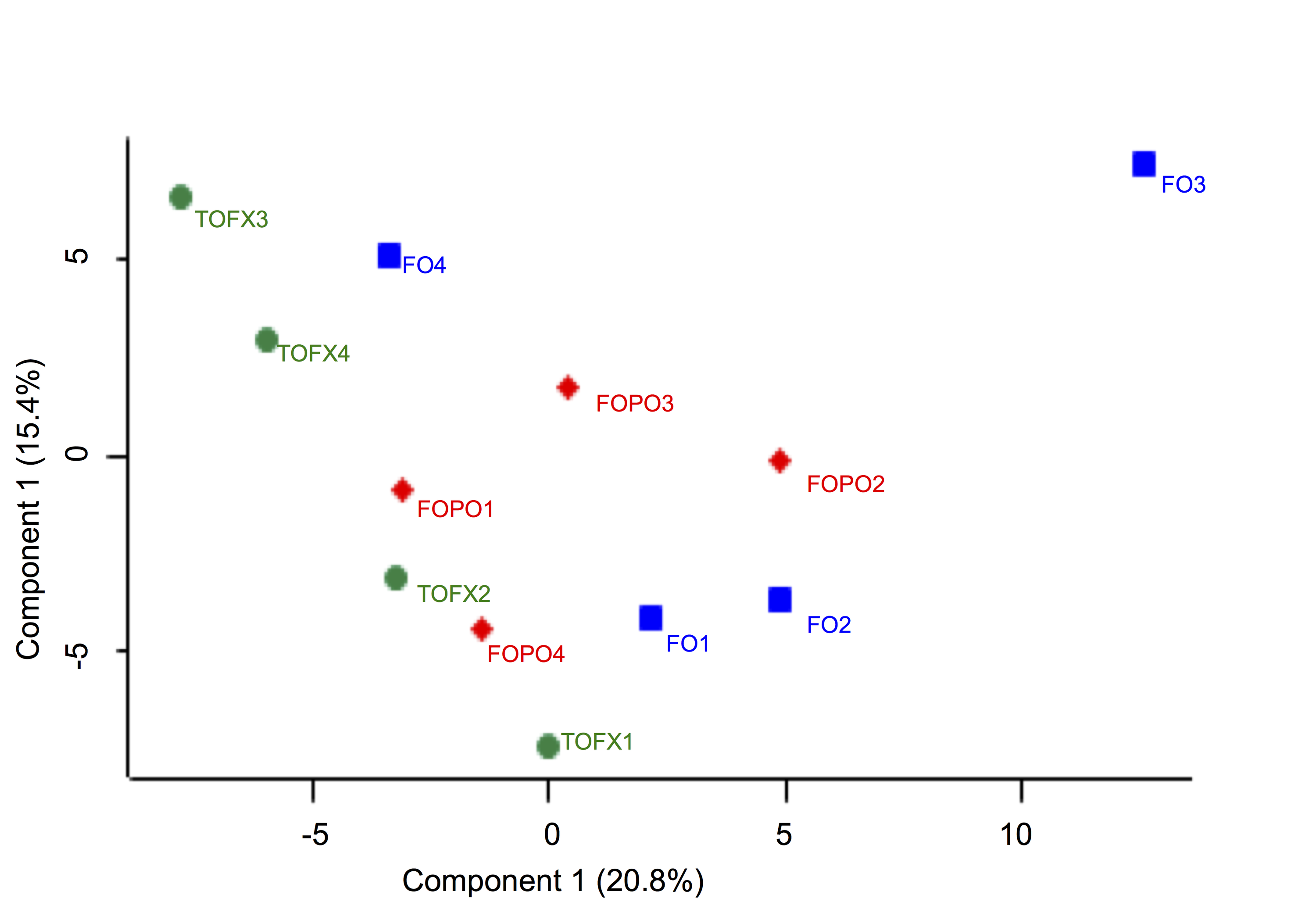

Supplement: S1 Fig — Data points are marked with sample identifiers and replicate number. (TIF) [file pone.0161513.s001.tif]
