# Supplementary material for: Preliminary Validation of a High Docosahexaenoic Acid (DHA) and -Linolenic Acid (ALA) Dietary Oil Blend: Tissue Fatty Acid Composition and Liver Proteome Response in Atlantic Salmon (Salmo salar) Smolts
Source: PLoS One. 2016 Aug 24;11(8):e0161513. doi: 10.1371/journal.pone.0161513 (PMC4996530; doi:10.1371/journal.pone.0161513)
Supplement: S1 Table — (DOCX) [file pone.0161513.s004.docx]

| **S1 Table. Apparent digestibility (AD; %) of fatty acids in Atlantic salmon smolt fed FO, FOPO and TOFX diets over a 89 day period** | | | |
| --- | --- | --- | --- |
|  |  |  |  |
|  | FO | FOPO | TOFX |
| 14:0 | 94.8 ± 0.90 | 95.9 ± 0.49 | 95.0 ± 0.67 |
| 16:0 | 89.7 ± 0.67 b | 90.1 ± 0.34 ab | 92.6 ± 0.59 a |
| 17:0 | 88.1 ± 0.75 a | 88.3 ± 0.66 a | 69.5 ± 2.17 b |
| 18:0 | 85.0 ± 0.90 | 84.8 ± 0.85 | 88.2 ± 0.87 |
| Total SFA | 90.2 ± 0.71 | 89.6 ± 0.41 | 91.2 ± 0.67 |
| 16:1n-7 | 99.2 ± 0.09 | 99.2 ± 0.13 | 99.2 ± 0.21 |
| 18:1n-7 | 97.7 ± 0.24 | 97.7 ± 0.32 | 98.2 ± 0.29 |
| 18:1n-9 (OA) | 98.1 ± 0.19 b | 98.7 ± 0.20 ab | 99.2 ± 0.20 a |
| 20:1n-9 | 95.8 ± 0.42 | 96.8 ± 0.92 | 95.0 ± 0.81 |
| 22:1n-11 | 95.9 ± 0.53 | 97.1 ± 0.36 | 95.3 ± 0.62 |
| Total MUFA | 97.9 ± 0.20 b | 98.5 ± 0.21 ab | 98.8 ± 0.22 a |
| 18:2n-6 (LA) | 97.7 ± 0.26 b | 99.1 ± 0.12 a | 99.0 ± 0.16 a |
| 18:3n-6 | 99.7 ± 0.15 | 99.6 ± 0.20 | 99.6 ± 0.23 |
| 20:3n-6 | 99.6 ± 0.22 | 98.8 ± 0.68 | 99.2 ± 0.42 |
| 20:4n-6 (ARA) | 98.8 ± 0.15 | 99.0 ± 0.13 | 98.4 ± 0.23 |
| Total n-6 PUFA | 98.2 ± 0.23 b | 99.1 ± 0.15 a | 98.9 ± 0.17 a |
| 18:3n-3 (ALA) | 98.8 ± 0.14 a | 99.1 ± 0.06 a | 96.5 ± 0.43 b |
| 18:4n-3 | 99.7 ± 0.05 | 99.6 ± 0.09 | 99.3 ± 0.19 |
| 20:4n-3 | 99.3 ± 0.15 | 99.0 ± 0.38 | 98.8 ± 0.34 |
| 20:5n-3 (EPA) | 99.6 ± 0.04 | 99.6 ± 0.10 | 99.5 ± 0.09 |
| 22:5n-3 | 99.2 ± 0.11 | 99.0 ± 0.31 | 98.6 ± 0.29 |
| 22:6n-3 (DHA) | 98.9 ± 0.13 a | 98.8 ± 0.18 a | 97.5 ± 0.39 b |
| Total n-3 LC PUFA | 99.3 ± 0.09 a | 99.1 ± 0.16 a | 98.5 ± 0.24 b |
| Total n-3 PUFA | 99.3 ± 0.08 a | 99.2 ± 0.14 a | 98.3 ± 0.21 b |
| Total PUFA | 99.3 ± 0.09 a | 99.2 ± 0.14 a | 98.6 ± 0.19 b |
| FO, oil content of feed is 100% fish oil; FOPO, oil content of feed is 20% fish oil and 80% poultry oil; TOFX, oil content of feed is 60% tuna oil and 40% flaxseed oil | | | |
|  |  |  |  |
|  |  |  |  |
| Data expressed as mean ± SEM (n=4). Different superscripts within a row denotes significant differences among diets as determined by Tukey-Kramer HSD (p<0.05) | | | |
|  |  |  |  |
|  |  |  |  |
